# Manipulating anion intercalation enables a high-voltage aqueous dual ion battery

**DOI:** 10.1038/s41467-021-23369-5

**Published:** 2021-05-25

**Authors:** Zhaodong Huang, Yue Hou, Tairan Wang, Yuwei Zhao, Guojin Liang, Xinliang Li, Ying Guo, Qi Yang, Ze Chen, Qing Li, Longtao Ma, Jun Fan, Chunyi Zhi

**Affiliations:** Department of Materials Science and Engineering, City University of Hong Kong, Kowloon, Hong Kong China

**Keywords:** Batteries, Energy, Batteries, Energy grids and networks, Batteries

## Abstract

Aqueous graphite-based dual ion batteries have unique superiorities in stationary energy storage systems due to their non-transition metal configuration and safety properties. However, there is an absence of thorough study of the interactions between anions and water molecules and between anions and electrode materials, which is essential to achieve high output voltage. Here we reveal the four-stage intercalation process and energy conversion in a graphite cathode of anions with different configurations. The difference between the intercalation energy and hydration energy of bis(trifluoromethane)sulfonimide makes the best use of the electrochemical stability window of its electrolyte and delivers a high intercalation potential, while BF_4_^−^ and CF_3_SO_3_^−^ do not exhibit a satisfactory potential because the graphite intercalation potential of BF_4_^−^ is inferior and the graphite intercalation potential of CF_3_SO_3_^−^ exceeds the voltage window of its electrolyte. An aqueous dual ion battery based on the intercalation behaviors of bis(trifluoromethane)sulfonimide anions into a graphite cathode exhibits a high voltage of 2.2 V together with a specific energy of 242.74 Wh kg^−1^. This work provides clear guidance for the voltage plateau manipulation of anion intercalation into two-dimensional materials.

## Abstract (web summary)

The interactions between water molecules, electrode materials and anions are essential yet challenging for aqueous dual ion batteries. Here, the authors demonstrate the voltage manipulation of dual ion batteries through matching intercalation energy and solvation energy of different anions.

## Introduction

The full use of renewable intermittent energy, such as wind, solar, and tidal energy, is a viable solution to meeting the growing global energy demand, which requires the development of large-scale energy storage technology with low cost, safety, and ecofriendliness^1–3^. Lithium-ion batteries (LIBs) possess satisfactory electrochemical performance and have been successfully commercialized, but the safety problems associated with the use of organic electrolytes and the expense of lithium salts and transition metals make them uneconomical grid-connected stationary energy storage systems (ESSs). In contrast, aqueous batteries have high safety and superior ion conductivity, and their assembly does not require an oxygen- and water-free environment, which could greatly reduce the cost of large-scale production. The renaissance of aqueous energy storage systems, especially the development of zinc ion energy storage technology provides a possible solution for stationary ESSs^4–6^, while the latest research indicates that the dendrites and corrosion of metal electrodes affect the cycling stability and cycle life^7,8^. Exploring a new aqueous battery system based on inexpensive components is pivotal to the development of large-scale ESSs.

**Fig. 1:**
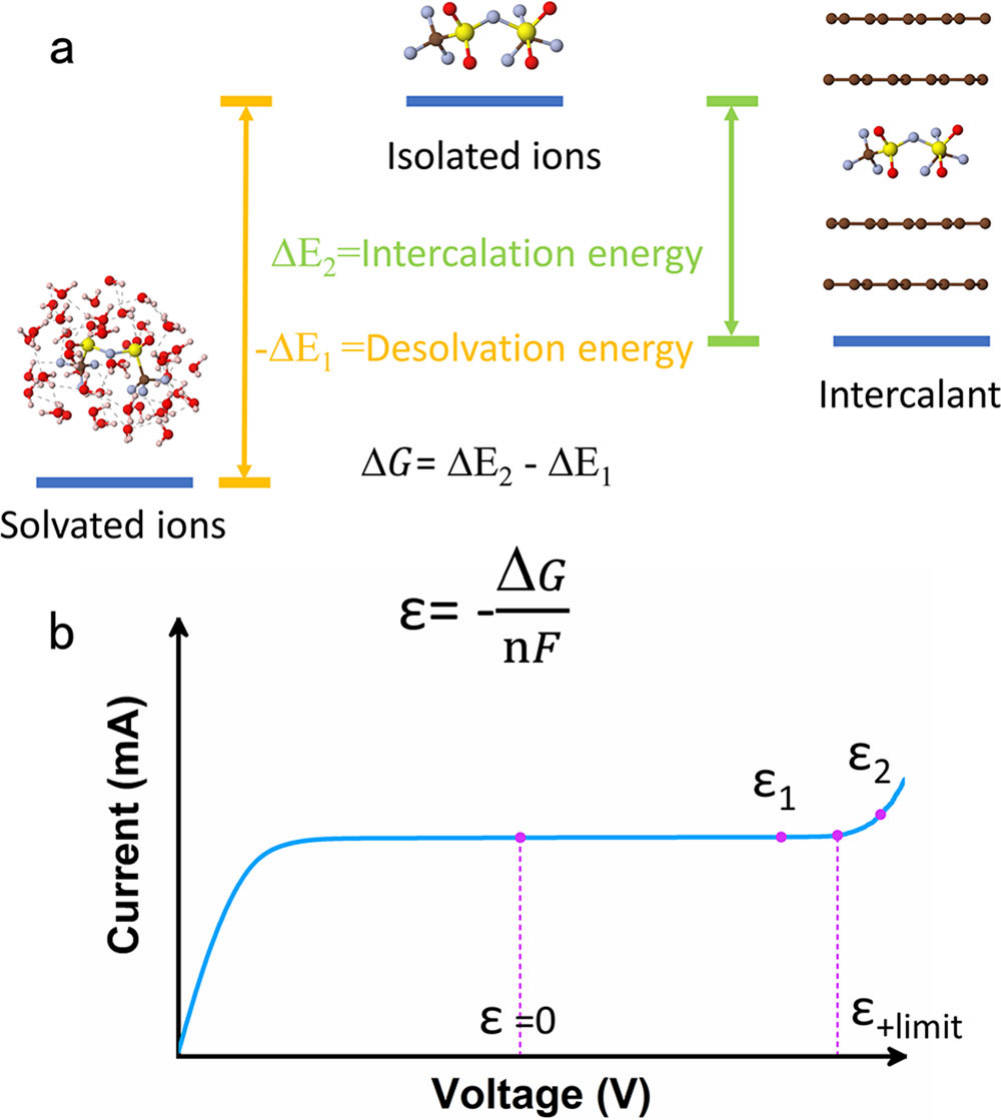
Regulation principles of high electrode potential. **a** Schematic of energy barriers between solvated ions, isolated ions, and intercalant state, corresponding to the desolvation process and intercalation process of anions insertion into graphite; **b** illustration exhibiting the electrode potential (ε) is calculated by Gibbs free energy (∆G) and should located within the electrochemical stability windows of electrolytes; n (mol) is the electron transfer number and F is the Faraday constant, ε_+limit_ is the cathode limit potential of electrolyte.

Graphite cathode-based dual ion batteries (DIBs) have attracted extensive attention due to their nontransition metal configuration^9–14^, which is economical and environmentally friendly, possessing an average high potential over 1.4 V vs. the standard hydrogen electrode (SHE)^15–19^. DIBs primarily differ from traditional rechargeable ion batteries in that they effectively introduce the intercalation behavior of anions and could greatly improve the output voltage of the batteries^20–22^. High-potential electrodes are highly important for improving the energy and specific power of rechargeable batteries. The output voltages of aqueous graphite-based DIBs depend on the intercalation energy and hydration energy, and thereby on the competition between the interaction of anions and the host matrix, as well as the interaction between anions and water. The energy barriers between solvated ions, isolated ions, and the intercalant state are shown in Fig. 1a. The energy difference between solvated ions and isolated ions is deemed the desolvation energy (denoted as −∆E_1_), corresponding to the desolvation process between ions inserted into the host matrix. The intercalation energy (denoted as ∆E_2_) is determined as the energy difference before the intercalant state and isolated ions. The electrode potential of graphite is determined as the Gibbs free energy difference between the intercalant and solvated ions (∆G = ∆E_2_−∆E_1_), according to the relationship between potential and Gibbs free energy (${{{\rm{\varepsilon }}}}=-\frac{\triangle {{{\rm{G}}}}}{{{{\rm{nF}}}}}$), which means that the electrode potential is positively correlated with the difference between intercalation energy and solvation energy. In addition, the electrode potential should be within the electrochemical stability window of the electrolyte (Fig. 1b, ε_1_ < ε_+limit_, where ε_+limit_ is the cathode limit potential of the electrolyte). Otherwise, the electrolyte will decompose before the anions are inserted into graphite (when ε_2_ > ε_+limit_). Hence, the key to achieving high voltages is to study in depth the interactions between anions and solvent as well as electrode materials. To explore the underlying mechanisms, a series of careful studies were conducted on the effects of anion intercalation energy and solvation energy on the voltage plateau of graphite cathode-based DIBs.

In this work, a high-voltage metal-free aqueous DIB was constructed with a 3,4,9,10-perylenetetracarboxylic diimide (PTCDI) organic anode and graphite cathode. The electrochemical behaviors of three types of anions, bis(trifluoromethanesulfonimide) (TFSI^−^), CF_3_SO_3_^−^ (Otf^−^), and BF_4_^−^, inserted into graphite were studied in three electrolytes (NaTFSI, NaOtf, and NaBF_4_). The intercalation energy and solvation energy were calculated using a modified Vienna ab initio simulation package (VASP) method and Gauss calculations based on first principles, through which the theoretical voltage plateaus of PTCDI-G DIBs in different electrolytes were determined. The optimized PTCDI-G DIB in the NaTFSI electrolyte delivered a high discharge voltage plateau of 2.2 V and a superior capacity of 120 mAh g^−1^. In addition, the energy storage mechanism of TFSI^-^ inserting into graphite in aqueous electrolytes has been thoroughly explored by in situ X-ray diffraction (XRD) and in situ Raman spectroscopy.

## Results

### Characterization of NaTFSI, NaOtf and NaBF_4_ electrolytes

**Fig. 2:**
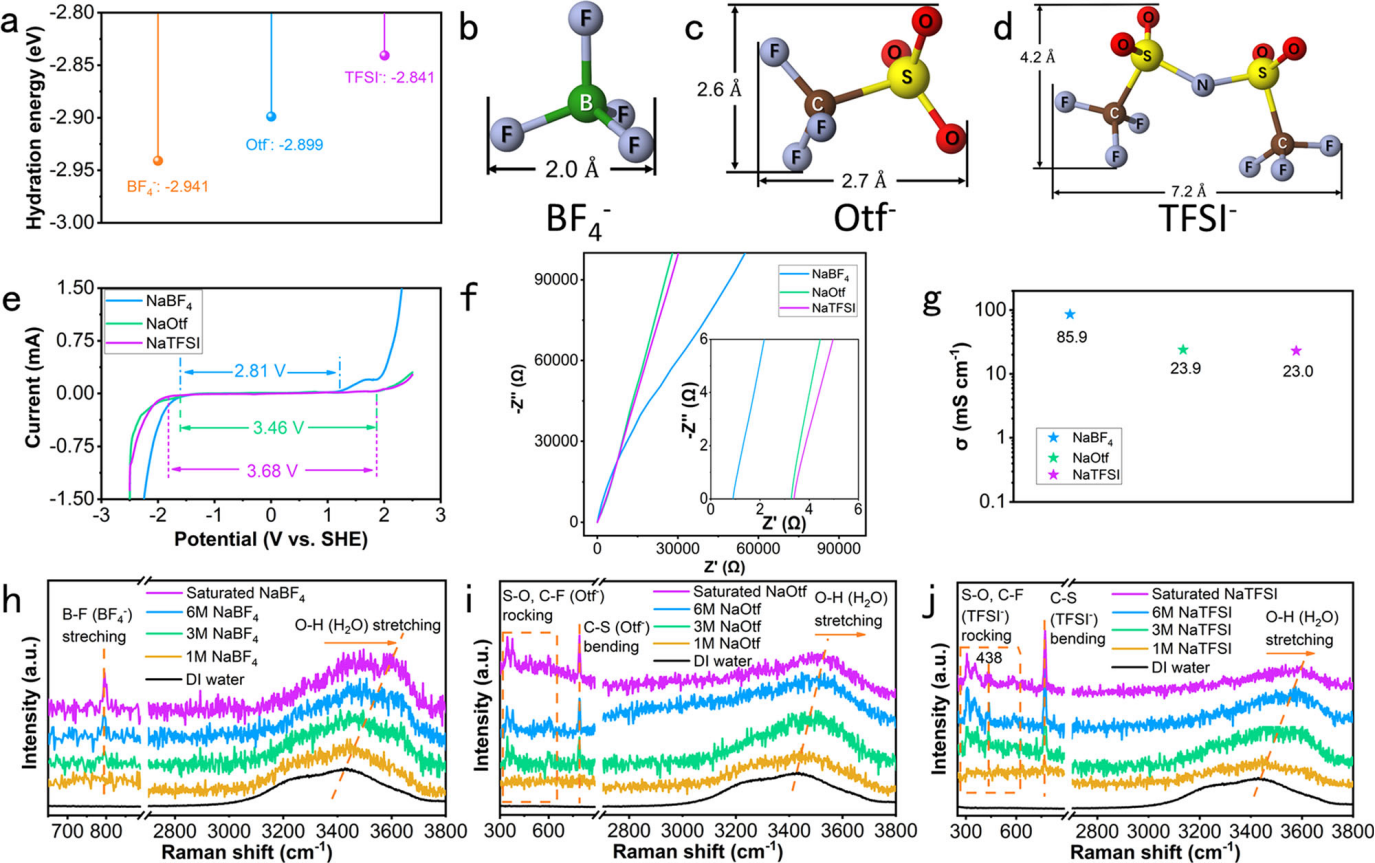
Physicochemical properties and Raman spectra of three electrolytes. **a** The hydration energy comparison of BF_4_^−^, Otf^−^, and TFSI^−^; (**b**) Diagrams of BF_4_^−^, (**c**) Otf^−^, and (**d**) TFSI^−^ anions, respectively, with length and width noted; (**e**) LSV curves of three electrolytes; (**f**) The EIS spectrum tested by a coin cell with Cu and SS foil as anode and cathode, respectively. The inset shows amplification of low-frequency areas; (**g**) The comparison of calculated ionic conductivity of three electrolytes; (**h**) Raman spectra of NaBF_4_, (**i**) NaOtf, and (**j**) NaTFSI electrolytes, respectively.

A large electrolyte mass is required for non-rocking-chair batteries as a reservoir of carriers, which entails maximizing the ionic content of the electrolyte. The use of highly concentrated salts not only widens the electrochemical stabilization windows of the electrolytes but also improves the specific energy of the DIBs. PF_6_^−^ anions are widely used in organic dual ion batteries, and their intercalation behaviors into graphite have been broadly studied. According to Feng’s report on Zn-graphite dual ion batteries^19^, the intercalation potential of PF_6_^−^ is lower than that of TFSI^−^. In addition, hexafluorophosphates are unstable in the air, which may pose a safety risk. Hence, considering the electrochemical performance and safety, we chose TFSI^−^, Otf^−^, and BF_4_^−^ anions to study the effects of anion type on the voltage of aqueous dual ion batteries. Generally, the lattice energy and the solvation energy of ions determine the solubility of a salt. The lattice energy represents the energy difference between the crystal lattice and isolated ions, which is semiquantitatively indicated by the lattice melting temperature^23^. The melting temperatures of NaTFSI, NaOtf, and NaBF_4_ are 257 °C, 254 °C, and 384 °C, respectively. The solvation energy of anions corresponds to the energy gained during the solvation process, which is positively correlated with the charge density of the anions^24,25^, i.e., the smaller the anions of a given charge are, the greater the solubility. The hydration energies of TFSI^−^, Otf^−^, and BF_4_^−^ were calculated through Gauss calculations based on first principles as shown in Fig. 2a, which will be discussed in detail later. The specific calculation method is provided in the Methods section. BF_4_^−^ showed the lowest hydration energy of −2.941 eV, and TFSI^−^ showed the highest hydration energy of −2.841 eV. The diagrams of the three anions (Fig. 2b–d) show that the widths of BF_4_^−^, Otf^−^, and TFSI^−^ were 2.0 Å, 2.6 Å, and 4.2 Å, respectively. High concentration saturated sodium salts (8.4 M NaTFSI, 9.2 M NaOtf, and 9.8 M NaBF_4_) were adopted as electrolytes because the effect of hydration energy on the solubility of these three salts is greater than that of the melting point.

The electrochemical stability windows of the three electrolytes were evaluated by linear sweep voltammetry (LSV) measurements, as displayed in Fig. 2e, with copper and steel stainless (SS) foils serving as anode and cathode current collectors, respectively. Because of the ultrahigh concentration of the three electrolytes, the distance between the cations and anions is enormously reduced, forcing them to share solvation molecules, so anions are inevitably involved in the primary solvation sheaths^26^. Thus, the content of water molecules in the sheath is depleted, which is beneficial for suppressing water-related parasitic reactions. In addition, ultrahigh concentrations of salt break the hydrogen bonding network between water molecules, which effectively decreases the mass transfer rate of H^+^ and OH^−^. Taking all these factors into consideration, the hydrogen and oxygen evolution reactions were efficiently inhibited and achieved a wide electrochemical stability window. The electrochemical stability windows of the NaBF_4_, NaOtf, and NaTFSI electrolytes are expanded to 2.81, 3.46, and 3.68 V, respectively. Notably, the cathode limits of NaBF_4_, NaOtf, and NaTFSI are 1.22 V, 1.88 V, and 1.88 V, respectively, which determined whether the anions could insert into graphite within the potential windows. The ionic conductivity of the electrolytes was evaluated by electrochemical impedance spectroscopy (EIS), as displayed in Fig. 2f. Coin cells with Cu and SS foil as the anode and cathode, respectively, were adopted for the EIS test. The inset of Fig. 2f shows that the x-intercepts of NaBF_4_, NaOtf, and NaTFSI were 0.9, 3.27, and 3.39 Ω. The ionic conductivities (σ, mS cm^−1^) were calculated using the following equation:

1 $$\sigma =\frac{{Z}^{{\prime} }}{{{Z}^{{\prime} }}^{2}+{{Z}^{{\prime} {\prime} }}^{2}}\bullet \frac{l}{A}$$

where *l* is the distance between two electrodes, which is approximately equal to the thickness of the separator, and *A* is the area of the separator. The ionic conductivities of NaBF_4_, NaOtf, and NaTFSI, as displayed in Fig. 2f, were calculated to be 85.9, 23.9, and 23.0 mS cm^−1^, respectively, which were positively correlated with their concentrations.

The Raman spectra of highly concentrated NaBF_4_, NaOtf, and NaTFSI electrolytes are shown in Fig. 2h–j. The characteristic peak at 795.7 cm^−1^ in Fig. 2h corresponds to the B–F symmetric stretching vibration of BF_4_^−^ in the solvent^27,28^, and increased in intensity with NaBF_4_ concentration. The O–H stretching vibration of H_2_O molecules showed an obvious blueshift with increasing salt concentration, which demonstrated the reduced activation of H_2_O in highly concentrated electrolytes^29–31^. The characteristic peaks from 300 to 650 cm^−1^ in the orange dotted box in Fig. 2i and j are attributed to the S=O and C–F rocking vibrations of Otf^−^ and TFSI^−^, while the peaks at ≈780 cm^−1^ can be attributed to the S=O and C–F asymmetric bending of Otf^−^ and TFSI^−^, respectively^32–34^. The extra peak located at 438 cm^−1^ in Fig. 2j is ascribed to the N–S–CF_3_ bending vibration of TFSI^−^^35,36^. Compared with those of deionized water, the O–H stretching vibrations of NaOtf and NaTFSI in the solution have an obvious blueshift trend as the concentration increases, indicating that the water activity is reduced because of the highly concentrated NaOtf and NaTFSI.

### Electrochemical performance comparison in three electrolytes

To construct metal-free electrode dual ion batteries, an organic electrode, PTCDI, was adopted as the anode material. The XRD and SEM images of PTCDI and graphite are shown in Supplementary Fig. 1. The electrochemical sodium storage performance of PTCDI in aqueous systems was explored through a three-electrode system with PTCDI, Pt, and Ag/AgCl serving as working, counter and reference electrodes, respectively. The sodium storage performance of the PTCDI anode may be affected by the Na^+^ concentration in the electrolyte but has no relationship with the type of anion, so saturated NaTFSI was adopted as the electrolyte. The CV and GCD curves of the PTCDI anode, shown in Supplementary Fig. 2, exhibited two distinct sodium ion extraction potentials at approximately −0.800 and 0.000 V vs. Ag/AgCl, corresponding to −0.5776 and 0.222 V vs. SHE. The initial five cycle CV curves in Supplementary Fig. 2a show that the first discharge process underwent a quasi-activation process, and the following four cycles almost completely overlapped each other, indicating the stable cycling ability of the PTCDI anode. GCD curves at different current densities from 0.25 A g^−1^ to 4 A g^−1^, as shown in Supplementary Fig. 2b, displayed a shape similar to the initial shape. Considering the superior sodium storage performance of PTCDI in aqueous electrolytes, it was matched with graphite to construct an aqueous sodium-based dual ion battery (PTCDI-G). The electrochemical properties of PTCDI-G were explored in three electrolytes including NaBF_4_, NaOtf, and NaTFSI saturated solutions, to study the effects of anion type and solvation energy on the electrochemical performance of dual-ion batteries, especially discharge voltage plateaus.

**Fig. 3:**
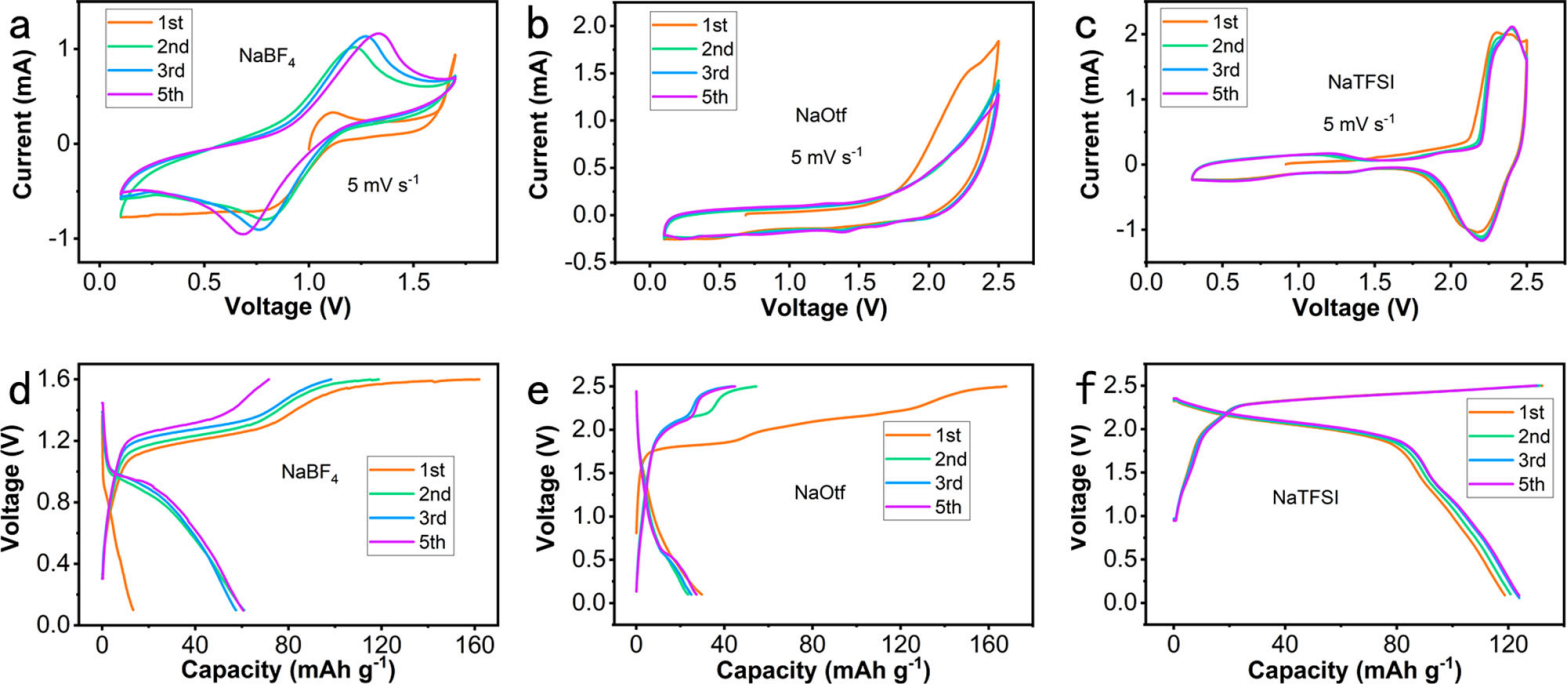
Electrochemical performance comparison of PTCDI-G in three electrolytes. **a** CV curves of PTCDI-G in NaBF_4_, (**b**) NaOtf, and (**c**) NaTFSI electrolytes at a scan rate of 5 mV s^−1^, respectively; (**d**) GCD curves of PTCDI-G in NaBF_4_, (**e**) NaOtf, and (**f**) NaTFSI electrolytes at a current density of 0.25 A g^−1^.

The CV and GCD curves of PTCDI-G in NaBF_4_, NaOtf, and NaTFSI electrolytes are compared in Fig. 3a–f. The initial five CV curves of PTCDI-G in the NaBF_4_ electrolyte are displayed in Fig. 3a. The initial anode curve showed a sharp increase in slope near 1.6 V, which could be ascribed to the partial decomposition of the electrolyte. This result was consistent with the charge plateau at 1.6 V while no distinct discharge plateau was observed in the first GCD curve, as shown in Fig. 3d. The subsequent CV curves showed a broad cathode peak between 0.7 and 1.0 V, which corresponded to the discharge plateau in the subsequent GCD curves. Figure 3b shows the initial five CV curves of the PTCDI-G DIB in the NaOtf electrolyte. There were strong anode peaks but no obvious cathode peaks, which was consistent with the GCD curves shown in Fig. 3e. Given that the sodium insertion and extraction potentials of the PTCDI anode were included in the electrochemical stability windows, it could be deduced that the potential of Otf^−^ embedded into graphite exceeded the cathode limit of the potential range. The CV curves and GCD curves of PTCDI-G in the NaTFSI electrolyte in the initial five cycles are displayed in Fig. 3c and f, which delivered an ultrahigh cathode peak (discharge plateau) at ≈2.2 V, as well as a superior capacity of 120 mAh g^−1^. Unlike those of the NaBF_4_ and NaOtf electrolytes, the CV and GCD curves of PTCDI-G in the NaTFSI electrolyte were coincident in the first and subsequent cycles, indicating its stable cycling performance. In addition, according to the GCD curves in Fig. 3f, the charge capacity remained similar, while the discharge capacity slowly increased with the cycling number, exhibiting the improved Coulombic efficiency of PTCDI-G in the NaTFSI electrolyte. The reasons for the differences in electrochemical behaviors among the three electrolytes will be discussed in detail later.

### In situ XRD and in situ Raman probing of TFSI^−^ intercalation

**Fig. 4:**
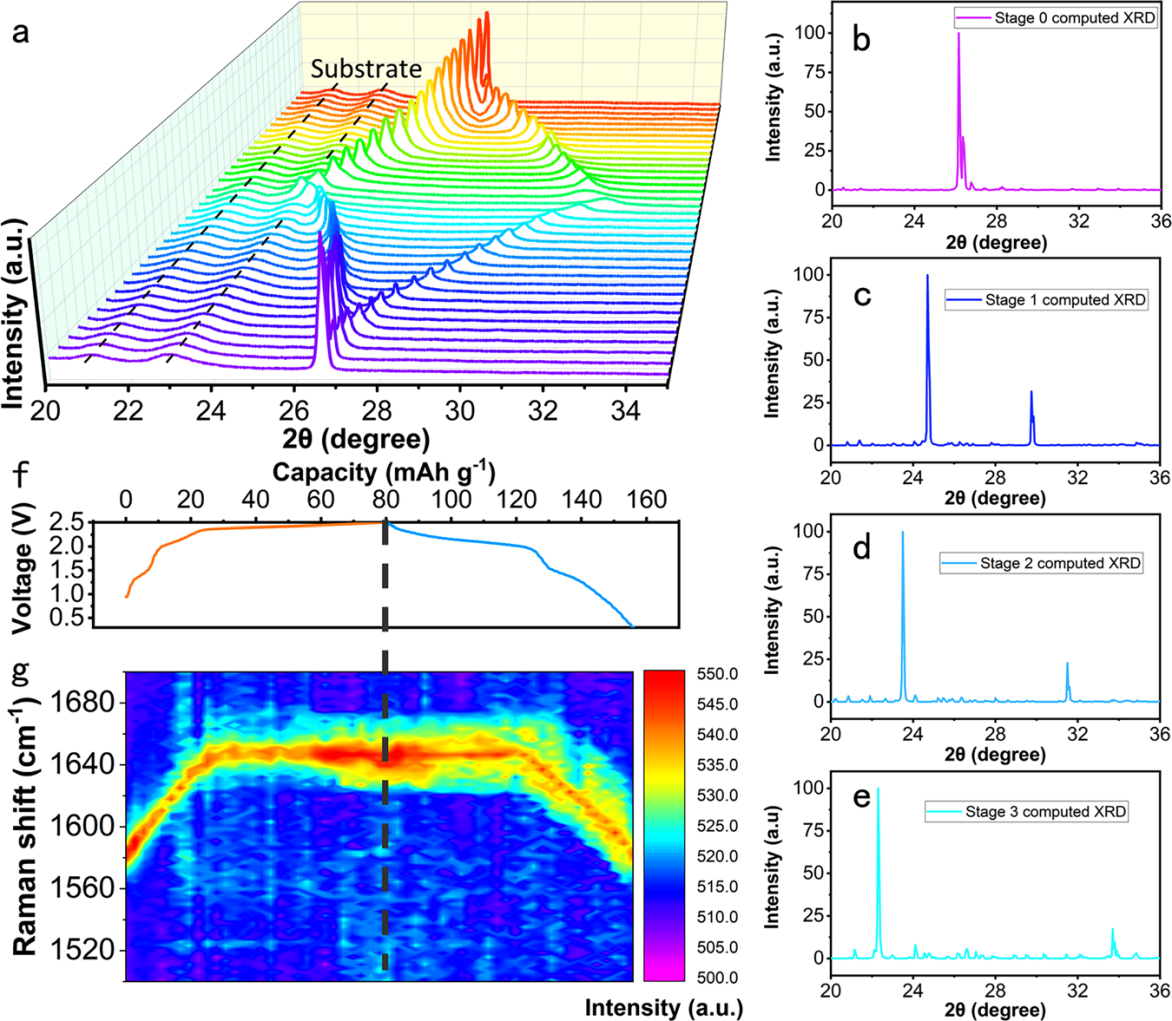
Energy storage mechanistic study of PTCDI-G in NaTFSI electrolyte. **a** In situ XRD patterns of graphite in PTCDI-G during the initial charging and discharging process; (**b**) The computed XRD of TFSI^−^ embedded into graphite at Stage 0, (**c**) Stage 1, (**d**) Stage 2, and (**e**) Stage 3, respectively; (**f**) The GCD curve tested at a current density of 50 mA g^−1^ for the initial cycle; (**g**) In situ Raman spectrum of graphite in PTCDI-G during the first charging and discharging process.

To investigate the energy storage mechanism of aqueous graphite-based DIBs, XRD and Raman measurements were conducted. Given that reaction products, such as graphite intercalation compounds (GICs), maybe decomposed after a series of coin cell detaching, washing, and drying processes, the use of in situ technology is necessary when studying energy storage systems involving GICs. The intercalation of complex anions embedded into graphite is in a unique, periodic way named the staging mechanism and, is caused by the balance between ionic repulsion of anions between the intercalant layers and van der Waals forces between graphite layers^37,38^. Typically, the staging mechanism proposed before comprised three stages from Stage 1 to Stage 3, which represent the insertion of anions in every four layers, two layers, and one layer of graphite, respectively. Another small plateau is observed at 1.5 V in NaTFSI electrolyte, far from the plateau of 2.2 V, which may be attributed to other energy storage behaviors such as adsorption behavior. Hence, we proposed a four-stage mechanism of complex anion insertion into graphite in aqueous electrolytes. A schematic diagram of the graphite intercalation process is displayed in Supplementary Fig. 3, in which Stage 0 corresponds to the edge plane adsorption state before the intercalation process. To verify the four-stage intercalation behaviors of anion insertion into the graphite cathode in the NaTFSI electrolyte, in situ XRD measurements were conducted. Figure 4a shows the in situ XRD patterns of the graphite cathode in PTCDI-G during the initial charging and discharging process at a current density of 50 mA g^−1^. The staging mechanism of TFSI^−^ intercalation into graphite can be demonstrated by the splitting of the (002) reflection during the charging process, signifying the formation of GICs^39–41^. The computed XRD patterns of TFSI^-^ anions embedded into graphite at different stages from Stage 0 to Stage 3 are provided in Fig. 4b–e, respectively. The variation in XRD profiles matched well with the computed XRD patterns including that of Stage 0, which verified that TFSI^−^ anions went through an edge plane adsorption state before the staging intercalation into graphite. Subsequently, the split peaks gradually returned to the original position of 26° as the anions were steadily extracted from graphite during the discharging process, demonstrating the high reversibility of anion insertion into/extraction from graphite.

In situ Raman characterization was also conducted to study the effects of TFSI^-^ anions on the vibration of hexagonal graphite. Figure 4g shows the real-time Raman signals at different voltages in the charging and discharging states from left to right, and Fig. 4f shows the corresponding GCD curve during Raman testing at a current density of 50 mA g^−1^. The original graphite showed a typical G band at 1580 cm^−1^, which corresponded to the strong carbon-carbon stretch vibration, representing the degree of graphitization^42^. The G band showed a blueshift with the continuous insertion of TFSI^−^ during the charging process, and finally shifted to 1650 cm^−1^ when charged to 2.5 V, demonstrating that the intercalated TFSI^−^ anions altered the chemical surroundings of the adjacent graphite layers, which was consistent with previous reports^43–45^. The Raman peaks subsequently showed a redshift and returned to the original position of graphite during the discharging process, further evidencing the good reversibility of TFSI^-^ intercalation into and extraction from graphite.

### First-principles calculation study of TFSI^−^, Otf^−^ and BF_4_^−^ anions intercalation into graphite

To better understand the relationship between anion intercalation behaviors based on a four-stage mechanism and the corresponding potentials, first-principles calculations were conducted. The ‘contact ion pair’ (CIP) model was adopted to calculate the theoretical voltage in previous reports^38,46^. This model assumes that dissolved salts remain in the form of contact ion pairs in the solvent and are surrounded by solvent molecules. We found it not applicable in our system, as the calculated results remarkably deviated from the experimental data, because it ignores the dissociation energy of the electrolyte molecule and the differences between the hydration energy of the CIP and that of the single anion. A more reliable approach is to calculate the solvation energy of anions separately in aqueous electrolytes when considering the solvation process of ions and anions and their hydrogen bonds with surrounding water molecules. The VASP and Gauss calculations based on first principles were adopted to calculate the intercalation energy and the solvation energy, respectively. The difference between the intercalation energy and the solvation energy is the theoretical voltage. Supplementary Table 1 displays the theoretical voltage and its difference with the experimental voltage as calculated using two models. The theoretical voltages of Otf^−^ calculated by the two models were ≈2.514 and 2.680 V, both of which exceeded the limit of electrochemical stability of the NaOtf electrolyte, which matched the experimental data that no discharge plateau was observed. However, the theoretical voltages calculated by applying the CIP model to the NaTFSI and NaBF_4_ electrolytes remarkably deviated from the experimental discharge voltage by up to 0.282 and 0.489 V, respectively. The differences between the theoretical voltages calculated through our model and the experimental voltages for TFSI^−^ and BF_4_^−^ were only 0.022 and 0.073 V, respectively, which matches well with practical GCD curves.

**Fig. 5:**
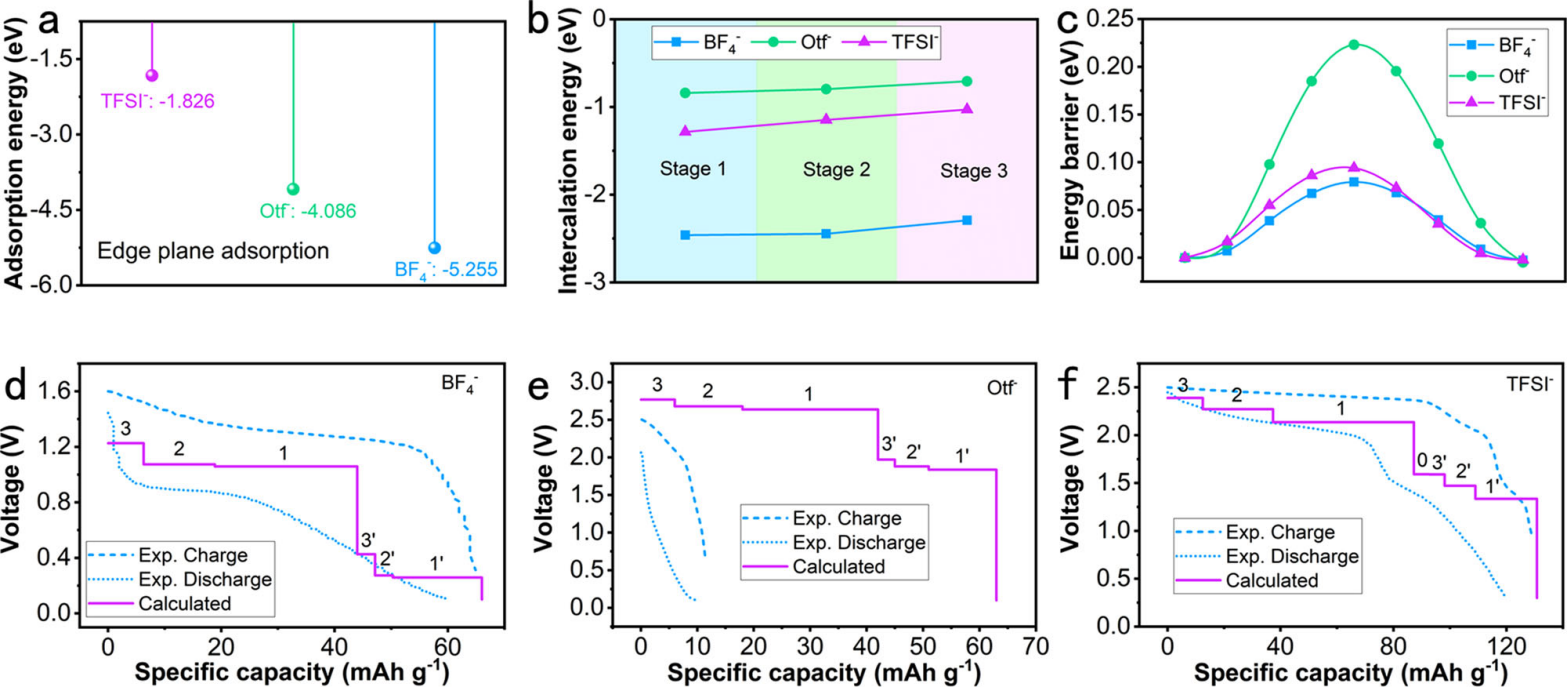
Theoretical calculations of PTCDI-G in three electrolytes and their corresponding calculated theoretical voltage. **a** The edge plane adsorption energy comparison for BF_4_^−^, Otf^−^, and TFSI^−^; (**b**) Intercalation energy of BF_4_^−^, Otf^−^, and TFSI^−^ embedded into graphite for Stage 1 to Stage 3; (**c**) The diffusion energy barrier of BF_4_^−^, Otf^−^, and TFSI^−^ in graphite matrix; (**d**) The calculated theoretical voltage and experimental voltages of PTCDI-G with NaBF_4_ electrolyte; (**e**) The calculated theoretical voltage and experimental voltages of PTCDI-G with NaOtf electrolyte; (**f**) The calculated theoretical voltage and experimental voltages of PTCDI-G with NaTFSI electrolyte. The marks of 0, 1, 2, 3 in (**d**–**f**) represent the calculated voltages of Stage 0 to Stage 3 matching with the first sodium extraction of PTCDI, respectively; the marks of 1’, 2’, 3’ represent the calculated voltages of Stage 1 to Stage 3 matching with the second sodium extraction of PTCDI, respectively.

To simulate the intercalation staging mechanism of the three anions, four stages from Stage 0 to Stage 3 are modeled. The edge plane adsorption of BF_4_^−^ on graphite is shown in Supplementary Fig. 4a–c, from which the decomposition of BF_4_^−^ was clearly observed. The tetrafluoroborate ion was converted to boron trifluoride, and another fluorine atom was bonded to graphite, which was ascribed to the greater stability of the carbon-fluorine bond compared to the boron-fluorine bond. The area of the edge plane was limited, which means that tetrafluoroborate ions partially decompose through an activation-like process before being embedded into graphite. This result can explain the oxidation peaks and decomposition plateaus at 1.6 V in Fig. 3a and d, leading to low Coulombic efficiency in the initial few cycles. The main view and top view of the subsequent insertion process from Stages 1–3 (see Supplementary Fig. 4d–i, respectively) show that three F atoms in the tetrafluoroborate ions lie directly above the three carbon atoms in a hexagonal graphite ring. The Otf^−^ anion was adsorbed on the edge plane of graphite through one of the O atoms bonding to graphite as displayed in Supplementary Fig. 5a–c. Because of the asymmetry of Otf^−^ anions and ionic repulsion between the adjacent intercalant, the Otf^−^ located in the adjacent layers rotated 90° as exhibited in Supplementary Fig. 5d–i, which greatly increased the migration barrier of ions. For TFSI^−^ anions, all four O atoms in TFSI^-^ have a strong tendency to bond with C atoms in graphite when adsorbed on the edge plane of graphite (see Supplementary Fig. 6a–c). Because of the symmetry of TFSI^−^ anions, the intercalants in different layers remained consistent with each other, as shown in Supplementary Fig. 6d–h. The adsorption energies of BF_4_^−^, Otf^−^, and TFSI^−^ on the edge plane of graphite are compared in Fig. 5a. BF_4_^−^ showed the lowest value of −5.255 eV, compared to −4.086 eV for Otf^−^ and −1.826 eV for TFSI^-^. The calculated intercalation energies of the three anions in the different stages are shown in Fig. 5b. BF_4_^−^ anions also possess the lowest intercalation energy in all stages due to their small radius and stronger C–F bond connected with graphite in the adsorbed state. The intercalation energies showed an increasing trend as the intercalation stage increased from Stage 1 to Stage 3: −2.46, −2.445, and −2.292 eV, respectively, because of ion repulsion between the intercalants. However, the Otf^−^ anions possessed the highest intercalation energy (−0.84, −0.797, −0.707 eV) in all stages due to their larger radius than that of BF_4_^−^ and the C–O bond being weaker than the C–F bond when absorbed. Similarly, the intercalation energies of TFSI^−^ were −1.283, −1.147, and −1.029 eV in Stage 1 to Stage 3, respectively, lower than that of Otf^−^, because four oxygen atoms of TFSI^−^ tended to be bonded with graphite when absorbed, compared to only two oxygen atoms of Otf^−^, as shown in Supplementary Fig. 5f and Supplementary Fig. 6f. Notably, PTCDI-G in the NaTFSI electrolyte showed four-stage intercalation behavior with a corresponding distinct voltage plateau, which was not observed in the NaOtf and NaBF_4_ electrolytes. Stage 0 corresponds to the edge plane adsorption behaviors on the graphite cathode. It depends on the adsorption energy and the solvation energy of anions in different electrolytes. The values of the difference between the adsorption energy and solvation energy for NaOtf and NaBF_4_ electrolytes were negative, which means no plateau corresponding to Stage 0 will appear when NaOtf or NaBF_4_ electrolytes are used.

The diffusion energy barriers of BF_4_^−^, Otf^−^, and TFSI^−^ in the graphite matrix are compared in Fig. 5c. The asymmetric structure of Otf^−^ anions increases the resistance to diffusion in the graphite matrix, leading to a large energy barrier (up to 0.223 eV). In contrast, the diffusion energy barriers of BF_4_^−^ and TFSI^−^ were always lower than 0.100 eV, enabling their fast diffusion during the electrochemical process.

The theoretical potential of anions inserted into graphite can be calculated through the intercalation energy and solvation energy of the anions, assuming that the contribution of entropic variation is negligible. The specific calculation method and equation are provided in the Methods section. The edge plane adsorption energies of BF_4_^−^ and Otf^−^ were much lower than their corresponding hydration energies, indicating that no output voltage plateau was contributed by their edge plane adsorption behaviors. In contrast, the adsorption energy and hydration energy of TFSI^-^ anions differed by 1.015 V; thus, the edge plane adsorption behaviors of TFSI^−^ anions would contribute a discharge plateau of 1.592 V combined with the first sodium extraction potential (−0.578 V vs. SHE) of the PTCDI anode. The intercalation potentials of BF_4_^−^ embedded in graphite through Stage 1 to Stage 3 were calculated as 0.481, 0.496, and 0.649 V vs. SHE, which were lower than the upper limit (1.22 V) of a highly concentrated NaBF_4_ electrolyte, as shown in Fig. 2e, demonstrating that the insertion of BF_4_^−^ anions into graphite can occur within the electrochemical stability window of the electrolyte. Considering the two sodium extraction potentials of the PTCDI anode (−0.5776 and 0.2224 V vs. SHE) and three intercalation stage potentials, there were six voltage plateaus for PTCDI-G in the NaBF_4_ electrolyte as displayed in Fig. 5d. The initial three voltage plateaus at 1.226, 1.073, and 1.058 V corresponded to the first sodium extraction potential of the anode, and the voltage plateaus at 0.426, 0.273 and 0.258 V corresponded to the second sodium extraction potential of PTCDI. For Otf^−^, the intercalation into graphite potentials was calculated to be 2.059, 2.102, and 2.192 V vs. SHE from Stage 1 to Stage 3, respectively. Considering that the electrochemical stability cathode limit of the NaOtf electrolyte was 1.88 V, the electrolyte decomposed before the Otf^−^ anions insert into the graphite cathode. This result means that the behavior of Otf^−^ anion intercalation into graphite was infeasible in highly concentrated NaOtf electrolyte, which is consistent with the previous discussion of CV and GCD results. The calculated theoretical voltages of PTCDI-G with the NaOtf electrolyte also have six plateaus at 2.770, 2.680, 2.637, 1.970, 1.880, and 1.837 V, respectively, as displayed in Fig. 5e, exhibiting a large gap with the experimental plateaus. Actually, the first charge curve shown in Fig. 3e exhibited a charging plateau at 1.8–1.9 V due to the partial insertion of Otf^−^ anions corresponding to the second sodium extraction potential of PTCDI, accompanied by the decomposition of the electrolyte. A voltage over 2 V corresponds to violent decomposition of the electrolyte, which is precisely why no corresponding discharging plateau was observed during the discharge process. The intercalation potentials of TFSI^-^ embedded into graphite were calculated to be 1.558, 1.694, and 1.812 V vs. SHE for Stage 1 to Stage 3, respectively, as shown in Fig. 5f. The potentials were lower than the cathode limit (1.88 V) of the NaTFSI electrolyte, enabling the insertion of TFSI^-^ anions into graphite within the electrochemical stability window of electrolyte. Considering the voltage plateau contributed by the edge plane adsorption behaviors, the calculated voltages of PTCDI-G in the NaTFSI electrolyte were expected to have seven voltage plateaus at 2.390, 2.272, 2.136, 1.592, 1.590, 1.472, and 1.336 V, respectively. Notably, the plateau provided by Stage 0 almost coincides with the plateau provided by Stage 1 corresponding to the first sodium extraction potential of PTCDI, which together contribute to the obvious discharge plateau at approximately 1.5 V of PTCDI-G with NaTFSI electrolyte. The interlayer spacing of the embedding layer and the average interlayer spacing after the intercalation of three anions are shown in Supplementary Table 2. The energy released by the stable intercalant formed by the insertion of anions is greater than the energy needed for the interlayer expansion of graphite; thus, the intercalation of anions leads to a large volume expansion of graphite. The interlayer spacings of fully charged graphite should be 7.27, 8.65, and 8.30 Å for BF_4_^−^, Otf^−^, and TFSI^−^ anions intercalation, respectively. The intercalation process of TFSI^−^ insertion into graphite is shown in Supplementary Movie 1, from which the expansion of graphite and the torsion of anions can be clearly observed.

The intercalation potentials of solvated anions inserted into host materials are determined by the solvation energy of the anions and the intercalation energy of the anions into the host. Supplementary Table 3 displays the intercalation potentials of different solvated anions, including TFSI^−^, Otf^−^, and BF_4_^−^, inserted into graphite at different intercalation stages.

The anion type mainly affects the potential of the solvated anions embedded into cathode materials, which depends on the solvation energy of the anions and the intercalation energy of the anions inserted into cathode host materials. The solvation energy corresponds to the energy gained by the anions during the solvation process, which is positively correlated with the charge density of the anions. The intercalation energy is determined by the interaction between the anions and host materials, as well as the size of the anions. For the insertion behaviors of anions into host materials, the more negative the solvation energy and the more positive the intercalation energy, the higher the theoretical cathode potential will be.

### Electrochemical performance of PTCDI-G in NaTFSI electrolyte

**Fig. 6:**
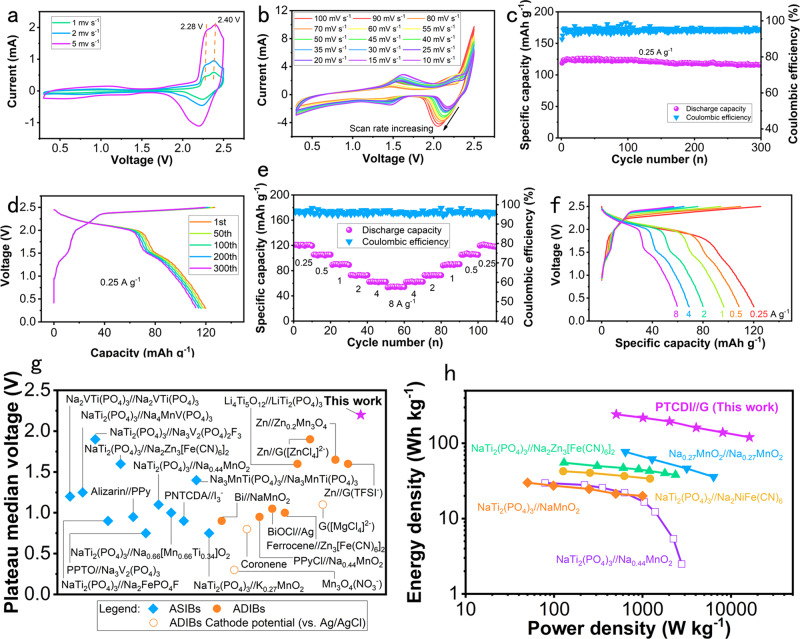
Electrochemical performance of PTCDI-G in NaTFSI electrolyte. **a** CV curves at relatively low scan rates of 1– 5 mV s^−1^; (**b**) CV curves at scan rates of 10 to 100 mV s^−1^; (**c**) Cycling performance at a current density of 0.25 A g^−1^; (**d**) GCD curves of different cycles; (**e**) Rate capability from the current density of 0.25 A g^−1^ to 8 A g^−1^; (**f**) GCD curves at different current densities; (**g**) The comparison of plateau median voltage between this work and previous reports about aqueous sodium ion batteries (ASIBs) and aqueous dual ion batteries (ADIBs); (**h**) Ragone plot of this work and other aqueous batteries (the calculation is based on the mass of graphite).

By comparing the electrochemical performance of the three electrolytes and the corresponding theoretical calculation, we found that the electrochemical performance of PTCDI-G in the NaTFSI electrolyte was better than those of the other methods. Therefore, we conducted a series of detailed tests on the electrochemical performance of PTCDI-G in the NaTFSI electrolyte. As shown in Fig. 6a, at relatively slow scan rates of 1, 2, and 5 mV s^−1^, two anode peaks appeared at 2.28 and 2.40 V, which coincided with the calculated voltages, demonstrating the staging mechanism of anion intercalation into graphite. The three discharging peaks coincided with each other, forming a broad cathode peak at ≈2.2 V. Figure 6b displays the fast scan rates from 10 mV s^−1^ to 100 mV s^−1^ of PTCDI-G, in which anode peaks located at 2.5 V and cathode peaks shifted from 2.2 V to 2.0 V because of the polarization at fast scan rates. The counter peaks at ≈1.5 V correspond to the edge plane adsorption behaviors and anion insertion behaviors and matched the second sodium extraction potential of PTCDI. Battery-like intercalation behaviors are usually considered diffusion-controlled processes, while adsorption on the surface of electrode materials is deemed a surface-controlled process. The adsorption behaviors on the surface of the electrode were surface-controlled behaviors, which contributed to a larger proportion of the capacitance as the scan rate increased. Hence, the redox peaks couple at 1.5 V obviously increases with the scan rate, which demonstrates the occurrence of edge plane adsorption behaviors of TFSI^−^ (Stage 0) from another perspective. The cycling performance of PTCDI-G is exhibited in Fig. 6c and delivered a capacity of 116.3 mAh g^−1^ after 300 cycles at a current density of 0.25 A g^−1^, with high-capacity retention of 98.0%. The corresponding GCD curves of different cycles from the 1st to 300th cycle are shown in Fig. 6d. The Coulombic efficiency of PTCDI-G in the NaTFSI electrolyte is approximately 95% upon long-term cycling. Inferior Coulombic efficiency is the common problem of graphite-based dual ion batteries and is primarily ascribed to the high voltage of graphite-based dual ion batteries and trace amounts of gas production. Electrolyte decomposition was accompanied by the charging process in the high voltage region. In addition, the low Coulombic efficiency was attributed to the oxidation and corrosion of edges of stainless-steel coin cells^37^. This problem should be solved by exploring aqueous electrolytes with wider electrochemical stability windows. The GCD curves almost coincided with each other, exhibiting the superior cycling stability of PTCDI-G. The rate performance of PTCDI-G in the NaTFSI electrolyte is shown in Fig. 6e, and delivered capacities of 121.0, 105.4, 90.3, 72.9, 62.4, and 54.8 mAh g^−1^ at current densities of 0.25, 0.5, 1.0, 2.0, 4.0, and 8.0 A g^−1^, respectively. When the current densities were set back to low densities, the capacity also returned to 62.5, 72.8, 90.2, 106.2, and 119.4 mAh g^−1^, respectively. The GCD curves at different current densities are displayed in Fig. 6f and exhibited similar shape and output voltage plateaus, demonstrating the good rate capability of PTCDI-G. The small output voltage plateau at ≈1.5 V became more obvious as the current density increased, which was consistent with the results of the CV curves in Fig. 5b. The long-term cycle life at high current density is shown in Supplementary Fig. 7. The PTCDI-G DIB in the NaTFSI electrolyte remained at 57 mAh g^−1^ after 10000 cycles at an ultrahigh current density of 8 A g^−1^. An activation process occurs during the initial several hundred cycles, which may be attributed to the insufficient intercalation of anions during the charging process at ultrahigh current density. The electrochemical performance of PTCDI-G DIB in NaTFSI electrolyte at different mass loadings is displayed in Supplementary Fig. 8. The GCD curves of PTCDI-G DIB with mass loadings of 1, 3, 5, 7, and 9 mg cm^−2^ are displayed in Figure S8a, and they delivered 0.12, 0.34, 0.53, 0.72, and 0.91 mAh of discharge capacity, respectively. In addition, all GCD curves showed an obvious high voltage plateau of 2.25 V. The linear fitting of capacity with different mass loadings is revealed in Figure S8b, among which the slope and intercept were 0.098 and 0.034, respectively.

The plateau median voltage of PTCDI-G in the NaTFSI electrolyte was compared with those of previous reports of aqueous sodium ion batteries (ASIBs)^47–58^ and aqueous dual ion batteries (ADIBs)^59–69^ as shown in Fig. 6g. Most of the voltage plateaus of ASIBs and ADIBs were lower than 1.5 V because of the narrow electrochemical stability windows of water. After the concept of ‘water in salt’ was proposed, many high voltage aqueous batteries have been explored. However, limited by the reactive potential of cathode materials, no aqueous batteries have a high plateau median voltage of over 2 V. The ultrahigh intercalation potential of TFSI^-^ embedded into graphite up to 1.812 V enabled the excited output voltage plateau of 2.2 V. More importantly, through the optimization of the intercalation anions and their hydration energy, the voltage of the anions inserted into two-dimensional materials still has great space for optimization. The specific power and specific energy of PTCDI-G were calculated based on the mass of graphite, and the specific calculation method is shown in the Methods section. PTCDI-G delivered high specific energy and specific power and was compared with other aqueous batteries, as exhibited in the Ragone plot (Fig. 6h). The PTCDI-G delivered high specific energy of 242.74 Wh kg^−1^ at 504.66 W kg^−1^, which remained at 120.41 Wh kg^−1^ when the specific power was increased to 16162.42 W kg^−1^.

## Discussion

We thoroughly investigated the influence of the interaction between anions and water and between anions and host materials on the electrochemistry of their reaction potentials to obtain a high-voltage aqueous DIB. TFSI^−^ can make the best use of the electrochemical stabilization window of its electrolyte to deliver a high intercalation potential due to its appropriate difference between intercalation energy and hydration energy. In contrast, the difference between the intercalation energy and hydration energy of BF_4_^−^ is small, and the graphite intercalation potential of Otf^−^ exceeds its electrolyte stability window, leading to their inferior output voltage. In addition, we revealed the energy conversion and four-stage intercalation behaviors of anions with different configurations in a graphite cathode. The one-to-one correspondence between the four stages of TFSI^−^ inserting onto graphite and their voltages was confirmed by in situ XRD and in situ Raman spectroscopy. The theoretical voltage plateaus calculated by DFT agree well with the experimental results, which further verified the feasibility of output voltage manipulation by studying the interaction among anions, water, and electrode materials. The DIB assembled with the PTCDI anode and graphite cathode based on the intercalation behaviors of TFSI^−^ delivered an ultrahigh voltage of 2.2 V, as well as a large capacity of 120 mAh g^−1^. This work is instructive for the design of high-voltage DIBs based on two-dimensional layered cathode materials.

## Methods

### Chemicals and battery components

Crystalline flake graphite (99.9%, Aladdin), 3,4,9,10-perylenetetracarboxylic diimide (PTCDI, 99.9%, Aladdin), sodium fluoroborate (NaBF_4_, 99.99%, Aladdin), sodium bis(trifluoromethanesulfonimide) (NaTFSI, battery grade, Huizhou Dado New Material Technology Co., Ltd), sodium trifluoromethanesulfonate (NaOtf, 98%, Aladdin), nmethyl-2-pyrrolidone (NMP, 99%, Aladdin), ketjenblack (ECP-600JD, Lion Corporation), poly(vinylidene fluoride) (PVDF, average Mw ~400000, Aladdin), Cu foil (9 μm, MTI Corporation), stainless foil (1 mm, MTI Corporation), and glass microfiber separator (GF/D-090, Whatman) were used as received.

### Preparation of saturated NaBF_4_, NaOtf, NaTFSI electrolytes

The electrolytes were prepared by adding 2.152 g NaBF_4_, 3.166 g NaOtf, 5.093 g NaTFSI into 2 mL deionized water, respectively. Transparent viscous liquids were formed after stirring vigorously.

### Assembly and testing of PTCDI-G with different electrolytes

The graphite cathode was prepared by mixing crystalline flake graphite, ketjenblack and PVDF (mass ratio: 8:1:1) in NMP solvent and stirred to form a black uniform slurry, followed by coating on stainless foil. The PTCDI anode was prepared by mixing PTCDI, ketjenblack and PVDF (mass ratio: 7:2:1) in NMP solvent and stirred to form a brown uniform slurry, followed by coating on Cu foil. Both the mass loading of graphite cathode and PTCDI anode are ca. 1 mg cm^−2^. The 2032-type coin cells of PTCDI-G batteries were assembled with graphite cathode and PTCDI anode (area ratio: 1:2) with a glass microfiber separator. These coin cells were cycled between 0.3 V and 2.5 V on CHI760E electrochemical workstation and LAND CT2001A battery testing devices. The specific energy and specific power were calculated based on the mass of graphite cathode through the following equations.

2 $$E\left({{{\rm{Wh}}}}{{{{\rm{kg}}}}}^{-1}\right)=\frac{I\left({{{\rm{A}}}}\right)\times V\left({{{\rm{V}}}}\right)\times t\left({{{\rm{h}}}}\right)}{m\left({{{\rm{kg}}}}\right)}$$

3 $$P\left({{{\rm{W}}}}{{{{\rm{kg}}}}}^{-1}\right)=\frac{E\left({{{\rm{Wh}}}}{{{{\rm{kg}}}}}^{-1}\right)}{t\left({{{\rm{h}}}}\right)}$$

where *I* is the charging and discharging current, *V* is the average output voltage, *t* is the discharging time, *m* is the mass of graphite cathode.

### Structural characterization of graphite

In situ XRD patterns were recorded with Cu Kα radiation (λ = 1.5406 Å) at 40 kV and 40 mA using Bruker AXS D8 Phaser. In situ and ex situ Raman spectra were performed with a laser of 532 nm wavelength using Raman spectroscopy (WITec alpha300 access). A Teflon electrochemical in situ cell with graphite and PTCDI as cathode and anode, respectively, were used for in situ XRD and Raman measurements. The CHI760E electrochemical workstation was utilized to record the GCD measuring when the XRD and Raman tests were performed.

### Computational methods

All the theoretical calculations were based on density functional theory (DFT) implemented in VASP^70^. The projector augmented wave (PAW) method was used to describe the interaction between electrons and ions. The exchange and correlation energy were described by gradient-corrected Perdew-Burke-Ernzerh (GGA-PBE)^71,72^ functional. According to the experimental results, the stage 3 configuration is set as C_24_[TFSI]^−^, C_24_[OTF]^−^ and C_36_[BF_4_]^−^. When optimizing the structure, a 520 eV cutoff of plane-wave basis was used. The first Brillouin zone was sampled by Monkhorst–Pack scheme with 0.06π/Å resolved value at each direction for all configurations. The Gaussian smearing with 0.2 eV smearing width was used. The accurate intercalation energy was further calculated by applying a tetrahedron method static self-consistency and 0.04 π/Å resolved Gamma-point mesh. The energy and force convergence criteria were set 1 × 10^−5^  eV and 0.01 eV/Å in structural optimization and energy calculation. The anion diffusion barrier at stage 1 was calculated by the climbing image nudged elastic band (NEB) method^73^ with 0.02 eV/Å force convergence criteria. To properly describe the van der Waals (vdWs) interaction, Grimme’s DFT-D3BJ empirical correlation was used^74,75^. And spin polarization was included in all calculations. The structure visualization was made by the VESTA software.

The intercalation voltage E_int_ can be approximately calculated as,

4 $${E}_{{int}}=\frac{E\left({{{{\rm{C}}}}}_{{{{\rm{n}}}}}{\left[{{{\rm{Anion}}}}\right]}_{{{{\rm{m}}}}}\right)-E\left({{{{\rm{C}}}}}_{{{{\rm{n}}}}}\right)-m{E}_{{gas}}\left({{{\rm{Anion}}}}\right)-m{E}_{{hyd}}\left({{{\rm{Anion}}}}\right)}{m}$$

In the formulation above, the entropic contribution is assumed as negligible, *E*(C_n_[Anion]_m_) is the total energy of anion-intercalated graphite, m is the number of intercalated anions, *E*(C_n_) is the energy of pure graphite in AB stacking, *E*_*gas*_(Anion) is the energy of the isolated anion in vacuum. *E*_*hyd*_(Anion) is the anion hydration energy calculated in Gaussian09 A.1 using SMD module^76^ with up to six explicit water molecules and B3LYP/6-311 G**^77,78^ with D3BJ empirical correlation^74,75^.

## Supplementary information

Supplementary information

Description of Additional Supplementary Files

Supplementary Movie 1

## Data Availability

The data that support the findings of this study are available from the corresponding author upon reasonable request.

## References

[1] Pomerantseva E (2019). Energy storage: the future enabled by nanomaterials. Science.

[2] Li Y (2019). Intercalation chemistry of graphite: alkali metal ions and beyond. Chem. Soc. Rev..

[3] Li M (2020). New concepts in electrolytes. Chem. Rev..

[4] Wang F (2018). Highly reversible zinc metal anode for aqueous batteries. Nat. Mater..

[5] Cao L (2020). Hydrophobic organic-electrolyte-protected zinc anodes for aqueous zinc batteries. Angew. Chem.,. Int. Ed..

[6] Jiang L (2019). Building aqueous K-ion batteries for energy storage. Nat. Energy.

[7] Yang Q (2019). Do zinc dendrites exist in neutral zinc batteries: a developed electrohealing strategy to in situ rescue in-service batteries. Adv. Mater..

[8] Ma L (2020). Realizing high zinc reversibility in rechargeable batteries. Nat. Energy.

[9] Yu M (2020). Interlayer gap widened alpha-phase molybdenum trioxide as high-rate anodes for dual-ion-intercalation energy storage devices. Nat. Commun..

[10] Qiao Y (2018). A hybrid electrolytes design for capacity-equivalent dual-graphite battery with superior long-term cycle life. Adv. Energy Mater..

[11] Sheng M (2017). A novel tin-graphite dual-ion battery based on sodium-ion electrolyte with high energy density. Adv. Energy Mater..

[12] Wang DY (2017). Advanced rechargeable aluminium ion battery with a high-quality natural graphite cathode. Nat. Commun..

[13] Zhang X (2016). A novel aluminum-graphite dual-ion battery. Adv. Energy Mater..

[14] Jiang L (2020). High-voltage aqueous na-ion battery enabled by inert-cation-assisted water-in-salt electrolyte. Adv. Mater..

[15] Chen Z, et al. Anion solvation reconfiguration enables high-voltage carbonate electrolytes for stable Zn/graphite cells. *Angew. Chem,. Int. Ed.* **59**, 21769–21777 (2020). 10.1002/anie.202010423 32812326

[16] Zhang J (2020). “Water-in-salt” polymer electrolyte for Li-ion batteries. Energy Environ. Sci..

[17] Liang G (2019). Commencing an acidic battery based on a copper anode with ultrafast proton-regulated kinetics and superior dendrite-free property. Adv. Mater..

[18] Tong X (2018). Core-shell aluminum@carbon nanospheres for dual-ion batteries with excellent cycling performance under high rates. Adv. Energy Mater..

[19] Wang G (2020). A high-voltage, dendrite-free, and durable Zn-graphite battery. Adv. Mater..

[20] Huang Z. et al. Effects of anion carriers on capacitance and self-discharge behaviors of zinc ion capacitor. *Angew. Chem. Int. Ed.* **60**, 1011–1021 (2021). 10.1002/anie.202012202 32965789

[21] Wang M (2018). Reversible calcium alloying enables a practical room-temperature rechargeable calcium-ion battery with a high discharge voltage. Nat. Chem..

[22] Liu Z (2019). Voltage issue of aqueous rechargeable metal-ion batteries. Chem. Soc. Rev..

[23] Gopal R (1955). Relation between lattice energy and melting points of some crystalline substances. II. Alkali Metals. Z. Anorg. Allg. Chem..

[24] Huang Z (2020). Phosphorene as Cathode Material for High-Voltage, Anti-Self-Discharge Zinc Ion Hybrid Capacitors. Adv. Energy Mater..

[25] Marcus Y (1991). Thermodynamics of solvation of ions. Part 5.—Gibbs free energy of hydration at 298.15 K. J. Chem. Soc., Faraday Trans..

[26] Suo L (2015). “Water-in-salt” electrolyte enables high-voltage aqueous lithium-ion chemistries. Science.

[27] Li X (2020). Vertically aligned Sn^4+^ preintercalated Ti_2_CTX MXene sphere with enhanced Zn ion transportation and superior cycle lifespan. Adv. Energy Mater..

[28] Quist AS (1971). Raman spectra of molten NaBF_4_ to 606 °C and 8% NaF-92% to 503 °C. J. Chem. Phys..

[29] Mo F., et al. An overview of fiber-shaped batteries with a focus on multifunctionality, scalability, and technical difficulties. Adv. Mater. 1902151 (2019). 10.1002/adma.201902151 31364216

[30] Chen J (2020). Pseudo-bonding and electric-field harmony for Li-rich mn-based oxide cathode. Adv. Funct. Mater..

[31] Yang Q (2020). Hydrogen-substituted graphdiyne ion tunnels directing concentration redistribution for commercial-grade dendrite-free zinc anodes. Adv. Mater..

[32] Zhu Y (2018). 3D interconnected ultrathin cobalt selenide nanosheets as cathode materials for hybrid supercapacitors. Electrochim. Acta.

[33] Liang G (2020). Initiating hexagonal MoO_3_ for superb-stable and fast NH^4+^ storage based on hydrogen bond chemistry. Adv. Mater..

[34] Huang Z (2017). Molybdenum phosphide: a conversion-type anode for ultralong-life sodium-ion batteries. Chem. Mater..

[35] Liu T. Electrodéposition de couches minces métalliques à partir de solutions de liquides ioniques pour des applications électroniques[D]. *Bordeaux* (2014).

[36] Li X (2020). In situ electrochemical synthesis of MXenes without Acid/Alkali usage in/for an aqueous zinc ion battery. Adv. Energy Mater..

[37] Schmuelling G (2013). X-ray diffraction studies of the electrochemical intercalation of bis(trifluoromethanesulfonyl)imide anions into graphite for dual-ion cells. J. Power Sources.

[38] Kravchyk KV (2018). High-energy-density dual-ion battery for stationary storage of electricity using concentrated potassium fluorosulfonylimide. Nat. Commun..

[39] Zhu J (2020). Hybrid aqueous/nonaqueous water-in-bisalt electrolyte enables safe dual ion batteries. Small.

[40] Xu X (2020). Quasi-solid-state dual-ion sodium metal batteries for low-cost energy storage. Chem.

[41] Chen Z (2020). Aqueous zinc-tellurium batteries with ultraflat discharge plateau and high volumetric capacity. Adv. Mater..

[42] Huang Z (2017). Layer-tunable phosphorene modulated by the cation insertion rate as a sodium-storage anode. Adv. Mater..

[43] Xiang L (2020). Highly concentrated electrolyte towards enhanced energy density and cycling life of dual-ion battery. Angew. Chem.,. Int. Ed..

[44] Lei X (2020). Highly stable magnesium-ion-based dual-ion batteries based on insoluble small-molecule organic anode material. Energy Storage Mater..

[45] Hu Z (2018). All carbon dual ion batteries. ACS Appl. Mater. Interfaces.

[46] Yang C (2019). Aqueous Li-ion battery enabled by halogen conversion-intercalation chemistry in graphite. Nature.

[47] Han J (2020). Halide-free water-in-salt electrolytes for stable aqueous sodium-ion batteries. Nano Energy.

[48] Ramesh Kumar P (2019). Na_4_MnV(PO_4_)_3_-rGO as advanced cathode for aqueous and non-aqueous sodium ion batteries. J. Power Sources.

[49] Liu S (2019). Na_3_V_2_(PO_4_)_2_F_3_–SWCNT: a high voltage cathode for non-aqueous and aqueous sodium-ion batteries. J. Mater. Chem. A.

[50] Sharma L (2019). Na_2_FePO_4_F fluorophosphate as positive insertion material for aqueous sodium-ion batteries. ChemElectroChem.

[51] Kühnel R-S (2017). A high-voltage aqueous electrolyte for sodium-ion batteries. ACS Energy Lett..

[52] Long H (2018). Self-assembled biomolecular 1D nanostructures for aqueous sodium-ion battery. Adv. Sci..

[53] Liang Y (2017). Universal quinone electrodes for long cycle life aqueous rechargeable batteries. Nat. Mater..

[54] Guo Z (2017). Multi-functional flexible aqueous sodium-ion batteries with high safety. Chem.

[55] Suo L (2017). “Water-in-Salt” electrolyte makes aqueous sodium-ion battery safe, green, and long-lasting. Adv. Energy Mater..

[56] Dong X (2016). Environmentally-friendly aqueous Li (or Na)-ion battery with fast electrode kinetics and super-long life. Sci. adv..

[57] Gao H (2016). An aqueous symmetric sodium-ion battery with NASICON-structured Na_3_MnTi(PO_4_)_3_. Angew. Chem. Int. Ed..

[58] Liu Y (2016). Hollow K0.27MnO2 nanospheres as cathode for high-performance aqueous sodium ion batteries. ACS Appl. Mater. Interfaces.

[59] Zhang Z (2018). Aqueous rechargeable dual-ion battery based on fluoride ion and sodium ion electrochemistry. J. Mater. Chem. A.

[60] Jiang H (2019). An aqueous dual-ion battery cathode of Mn_3_O_4_ via reversible insertion of nitrate. Angew. Chem. Int. Ed..

[61] Rodríguez-Pérez IA (2019). Aqueous anion insertion into a hydrocarbon cathode via a water-in-salt electrolyte. Electrochem. Comm..

[62] Kong H (2019). Polypyrrole as a novel chloride-storage electrode for seawater desalination. Energy Technol..

[63] Zhang Z (2019). The composite electrode of Bi@carbon-texture derived from metal-organic frameworks for aqueous chloride ion battery. Ionics.

[64] Wu X (2019). Reverse dual-ion battery via a ZnCl_2_ water-in-salt electrolyte. J. Am. Chem. Soc..

[65] Wrogemann JM (2020). Development of safe and sustainable dual-ion batteries through hybrid aqueous/nonaqueous electrolytes. Adv. Energy Mater..

[66] Guo Q. et al. A high-potential anion-insertion carbon cathode for aqueous zinc dual-ion battery. *Adv. Funct. Mater*. **30**, 2002825 (2020).

[67] Kim K. I. et al. Reversible insertion of Mg-Cl superhalides in graphite as a cathode for aqueous dual-ion batteries. *Angew. Chem., Int. Ed.* **132**, 20096–20100 (2020). 10.1002/anie.202009172 32710468

[68] Jiang H (2020). Counter-ion insertion of chloride in Mn_3_O_4_ as cathode for dual-ion batteries: a new mechanism of electrosynthesis for reversible anion storage. Carbon Energy.

[69] Zhang H (2020). Electrochemical intercalation of anions in graphite for high-voltage aqueous zinc battery. J. Power Sources.

[70] Kresse G (1996). Efficient iterative schemes for ab initio total-energy calculations using a plane-wave basis set. Phys. Rev. B.

[71] Blochl PE (1994). Improved tetrahedron method for Brillouin-zone integrations. Phys. Rev. B.

[72] Perdew JP (1996). Generalized gradient approximation made simple. Phys. Rev. Lett..

[73] Henkelman G (2000). A climbing image nudged elastic band method for finding saddle points and minimum energy paths. J. chem. phys..

[74] Grimme S (2010). A consistent and accurate ab initio parametrization of density functional dispersion correction (DFT-D) for the 94 elements H-Pu. J. Chem. Phys..

[75] Grimme S (2011). Effect of the damping function in dispersion corrected density functional theory. J. Comput. Chem..

[76] Marenich AV (2009). Universal solvation model based on solute electron density and on a continuum model of the solvent defined by the bulk dielectric constant and atomic surface tensions. J. Phys. Chem. B.

[77] Stephens PJ (1994). Ab initio calculation of vibrational absorption and circular dichroism spectra using density functional force fields. J. Comput. Chem..

[78] McLean AD (1980). Contracted Gaussian basis sets for molecular calculations. I. Second row atoms, Z=11–18. J. Chem. Phys..

