# Author Correction: Manipulating anion intercalation enables a high-voltage aqueous dual ion battery

**DOI:** 10.1038/s41467-021-25179-1

**Published:** 2021-08-06

**Authors:** Zhaodong Huang, Yue Hou, Tairan Wang, Yuwei Zhao, Guojin Liang, Xinliang Li, Ying Guo, Qi Yang, Ze Chen, Qing Li, Longtao Ma, Jun Fan, Chunyi Zhi

**Affiliations:** Department of Materials Science and Engineering, City University of Hong Kong, Kowloon, Hong Kong China

**Keywords:** Batteries, Energy, Batteries, Energy grids and networks, Batteries

Correction to: *Nature Communications* 10.1038/s41467-021-23369-5, published online 25 May 2021.

The original version of this Article did not acknowledge Prof. Jun Fan as a corresponding author. This has now been corrected in both the PDF and HTML versions of the Article.

