# Supplementary material for: Manipulating anion intercalation enables a high-voltage aqueous dual ion battery
Source: Nat Commun. 2021 May 25;12:3106. doi: 10.1038/s41467-021-23369-5 (PMC8149852; doi:10.1038/s41467-021-23369-5)
Supplement: Supplementary file 1 — Supplementary information [file 41467_2021_23369_MOESM1_ESM.pdf]

Supporting information

Manipulating anions intercalation enables a high-voltage aqueous dual ion battery

Zhaodong Huang, Yue Hou, Tairan Wang, Yuwei Zhao, Guojin Liang, Xinliang Li, Ying Guo, Qi Yang, Ze Chen, Qing Li, Longtao Ma, Jun Fan*, Chunyi Zhi*

Department of Materials Science and Engineering, City University of Hong Kong, 83 Tat Chee Avenue, Kowloon, Hong Kong 999077, China. E-mail: cy.zhi@cityu.edu.hk (C. Zhi).

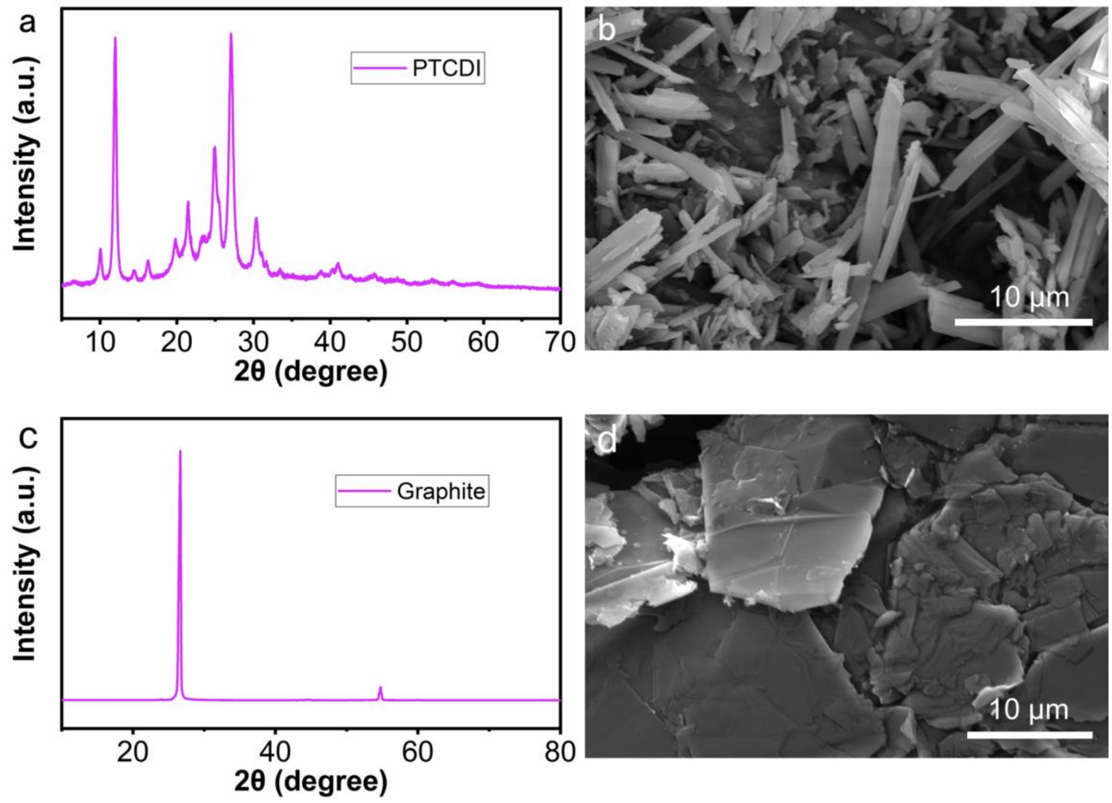

Supplementary Fig. 1 a, b XRD pattern and SEM image of PTCDI; **c, d** XRD pattern and SEM image of graphite.

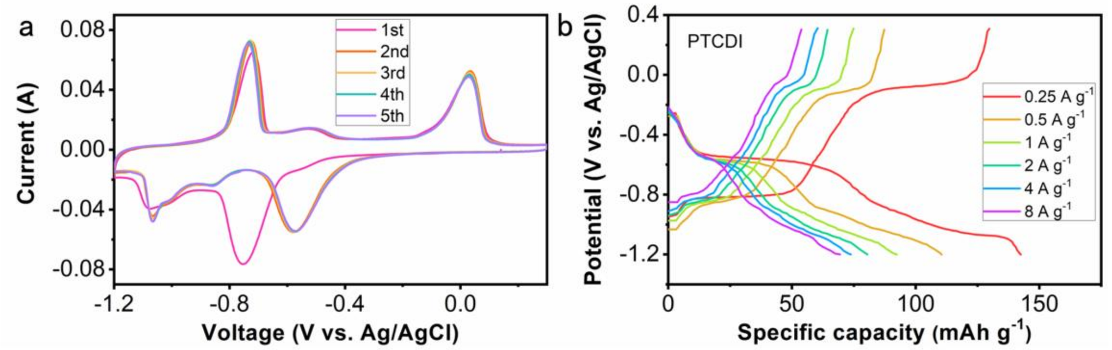

Supplementary Fig. 2 a Three electrode CV curves in initial five cycles and **b** GCD curves at different current densities. The PTCDI, Pt and Ag/AgCl were adopted as working, counter and reference electrodes, respectively, with saturated NaTFSI as electrolyte.

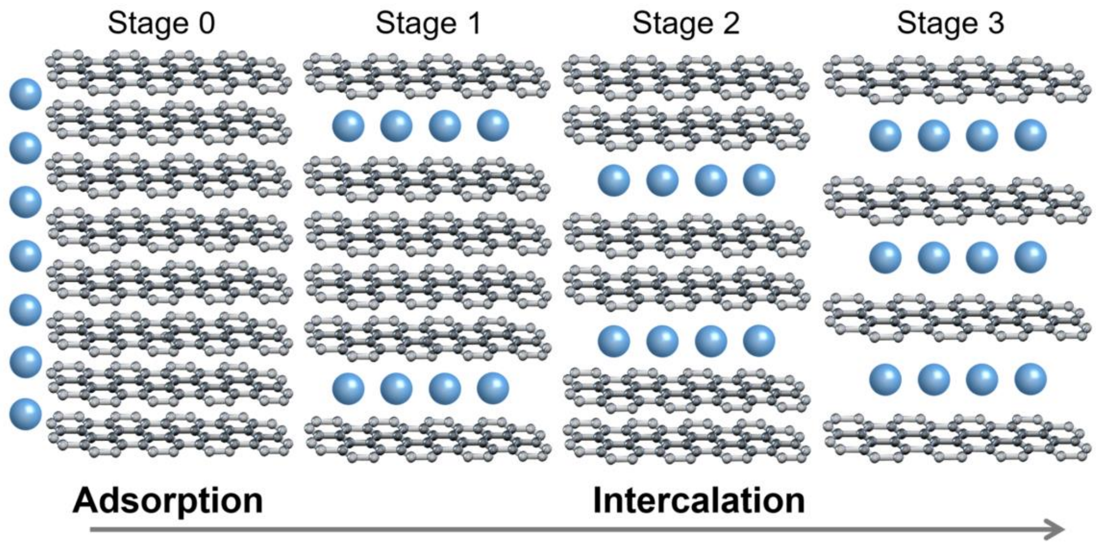

Supplementary Fig. 3 Schematic diagram of graphite intercalation staging mechanism from Stage 0 to Stage 3.

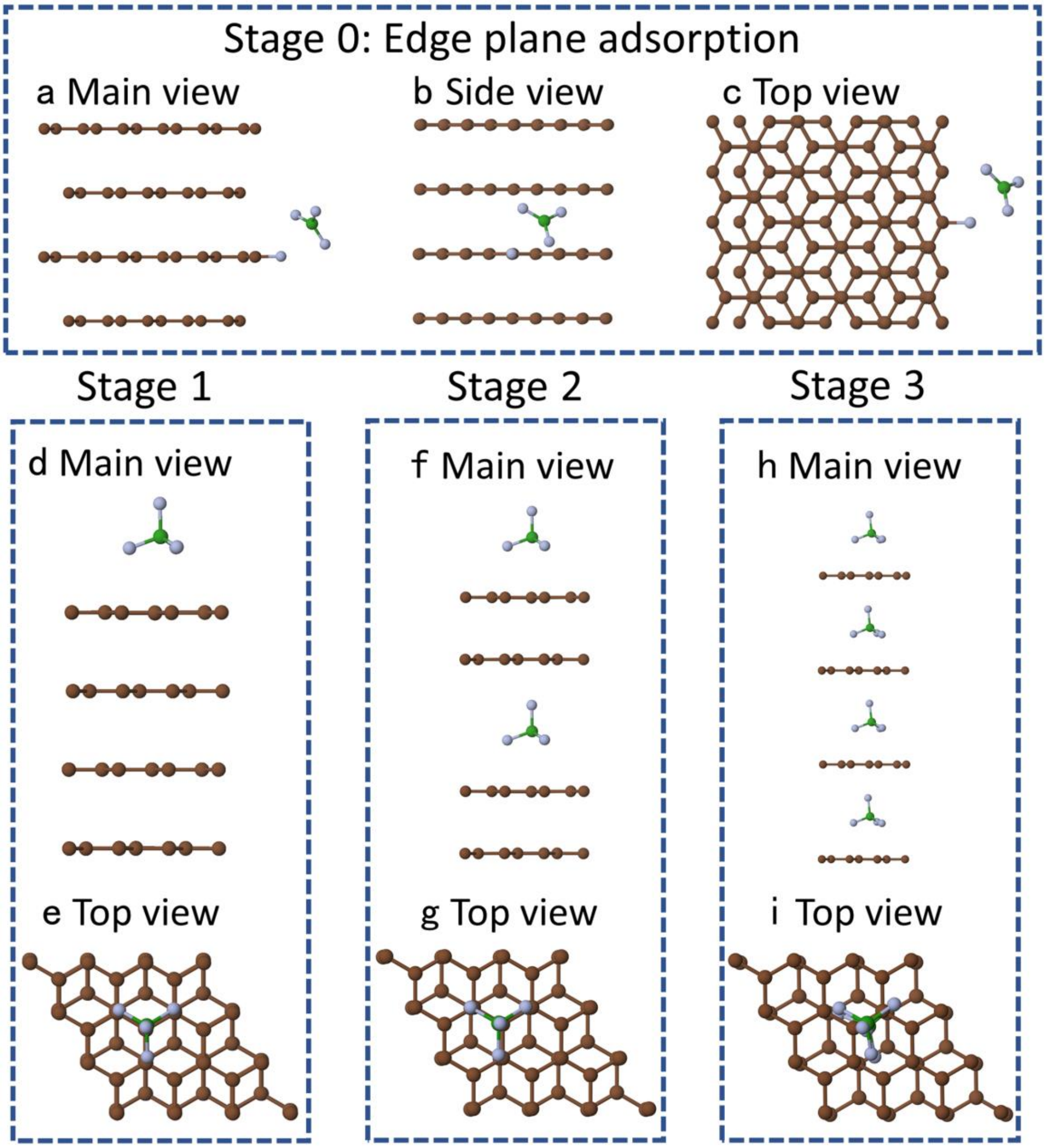

Supplementary Fig. 4 **a** Main view, **b** side view and **c** top view of edge plane adsorption of BF_4^- on graphite; The main view and top view of following insertion process from **d, e** Stage 1, **f, g** Stage 2 and **h, i** Stage 3, respectively.

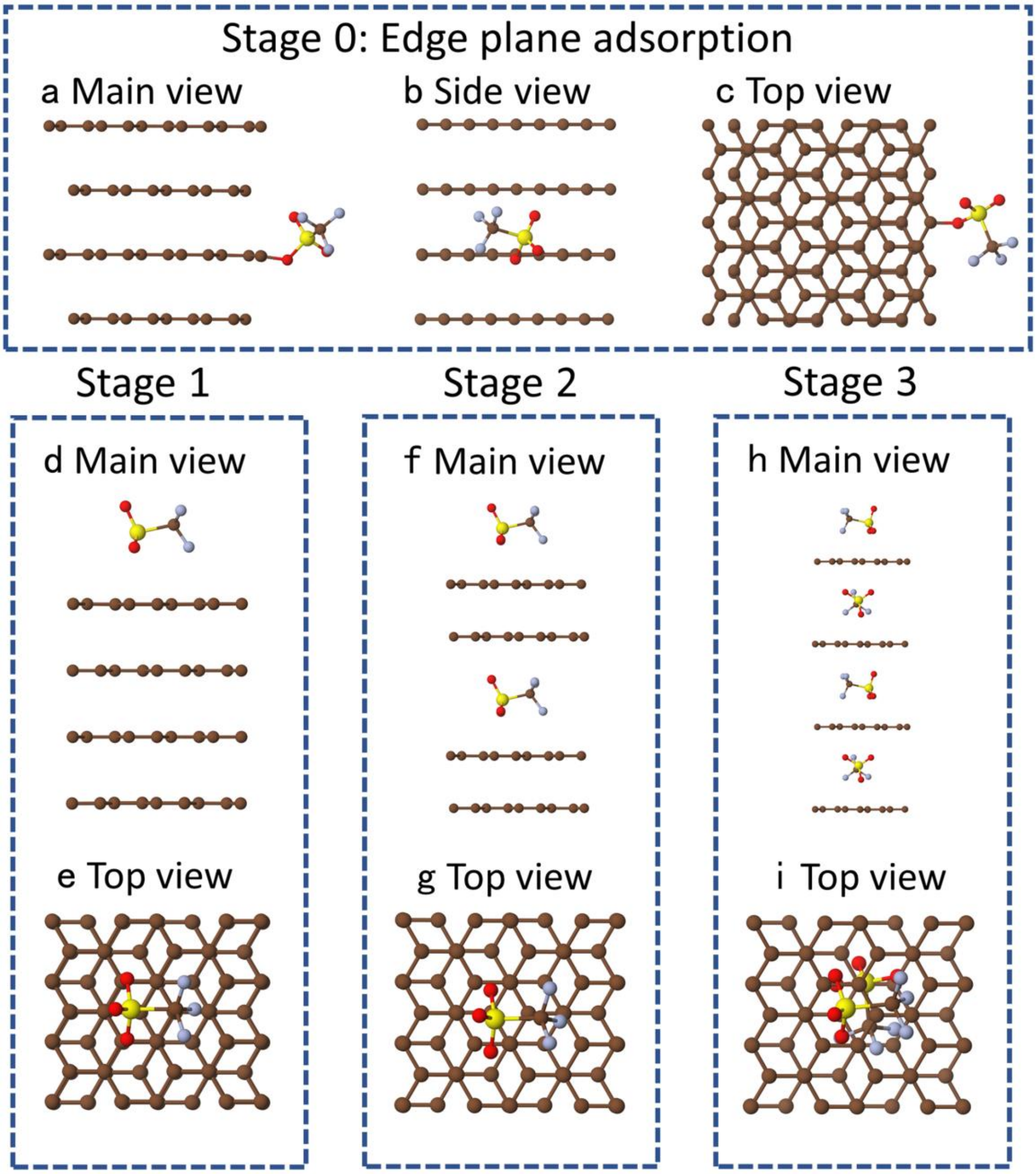

Supplementary Fig. 5 a Main view, **b** side view and **c** top view of edge plane adsorption of Otf$^-$ on graphite; The main view and top view of following insertion process from **d, e** Stage 1, **f, g** Stage 2 and **h, i** Stage 3, respectively.

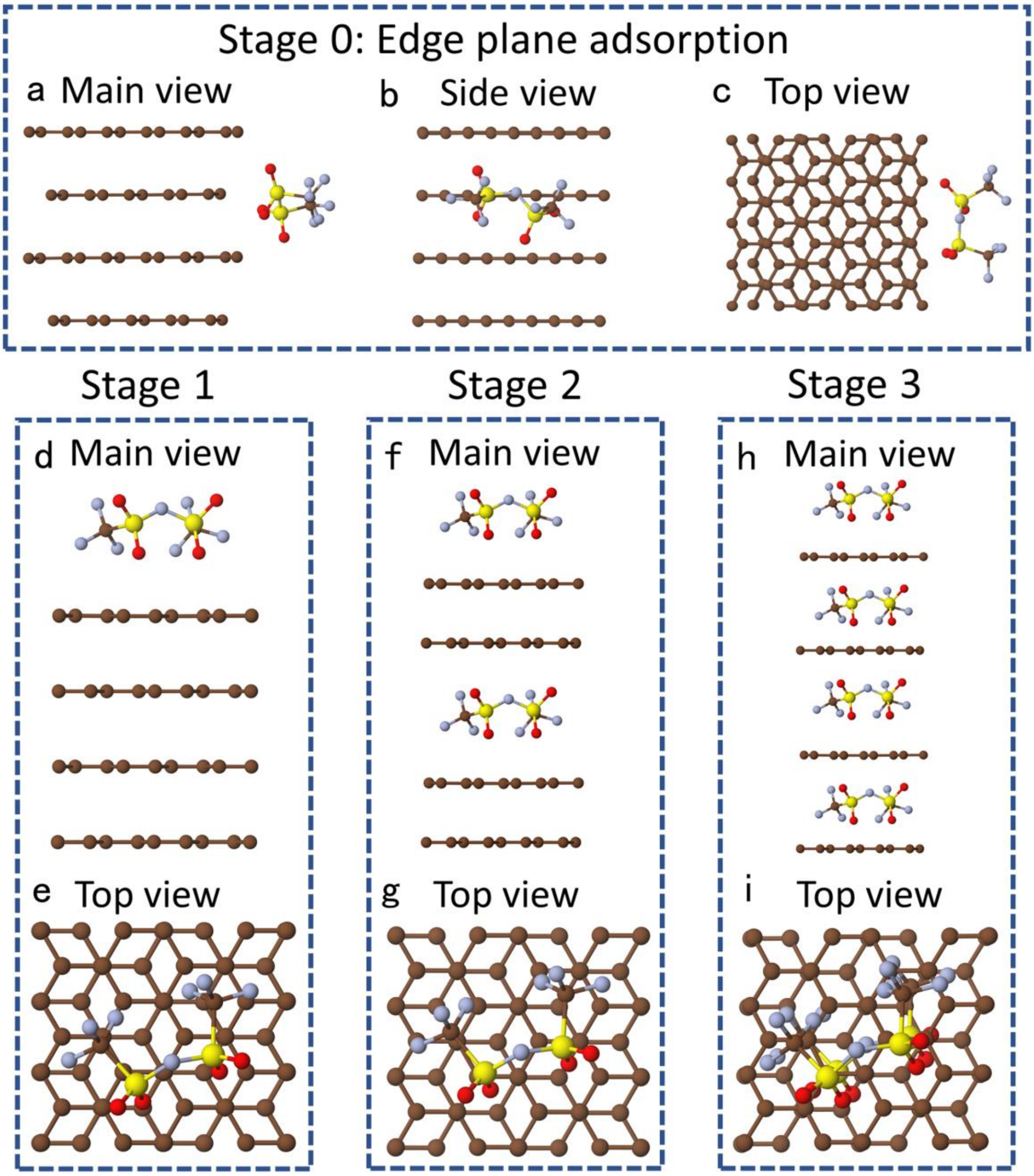

Supplementary Fig. 6 a Main view, **b** side view and **c** top view of edge plane adsorption of TFSI⁻ on graphite; The main view and top view of following insertion process from **d, e** Stage 1, **f, g** Stage 2 and **h, i** Stage 3, respectively.

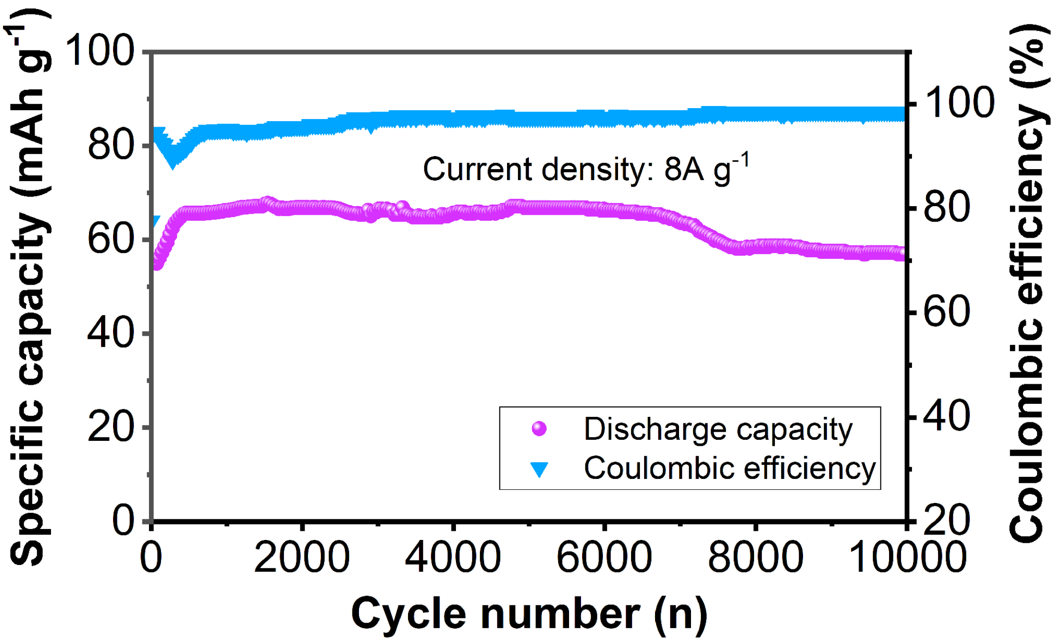

Supplementary Fig. 7 Cycling performance of PTCDI-G in NaTFSI electrolyte at ultrahigh current density of 8A g^{-1}.

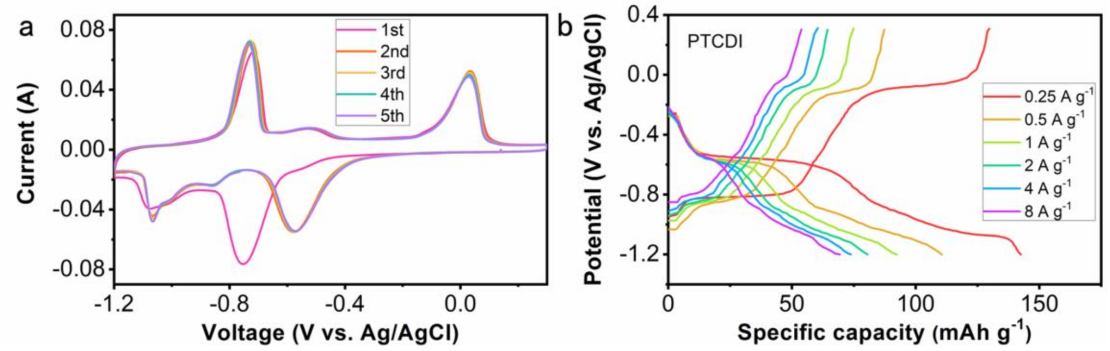

Supplementary Fig. 8 a The GCD curves of PTCDI-G DIB in NaTFSI electrolyte at different mass loadings; **b** The linear fitting of capacity with different mass loadings.

Supplementary Table 1. The comparison of theoretical voltage of PTCDI-G in NaTFSI electrolyte calculated by CIP model and our model.

Anions	Stages	Experimental voltage (V)	Calculated voltage (V, through CIP model)	Differences (V)	Calculated voltage (V, through our model)	Differences (V)
TFSI-	Stage 1	2.25 V (average)	1.832	-0.282	2.136	0.022
	Stage 2		1.968		2.272	
	Stage 3		2.086		2.39	
Otf-	Stage 1	/	2.471	/	2.637	/
	Stage 2		2.514		2.68	
	Stage 3		2.604		2.77	
BF4-	Stage 1	1.0 V (average)	1.474	0.489	1.058	0.073
	Stage 2		1.489		1.073	
	Stage 3		1.642		1.226	

Supplementary Table 2. The interlayer spacing of the embedding layer and average interlayer spacing for graphite after the intercalation of BF_4^-, Otf^- and $TFSI^-$ anions.

	The interlayer spacing of the embedding layer (angstrom, Å)			Average interlayer spacing (angstrom, Å)		
Stage	S1	S2	S3	S1	S2	S3
$TFSI^-$	7.97	8.00	8.30	4.50	5.69	8.30
Otf^-	8.53	8.57	8.65	4.65	5.98	8.65
BF_4^-	7.12	7.15	7.27	4.30	5.25	7.27

Supplementary Table 3. The intercalation potentials of different solvated anions inserted into graphite.

Anions	Stages	Cathode potential (V vs. SHE)
TFSI-	Stage 1	1.558
	Stage 2	1.694
	Stage 3	1.812
Otf-	Stage 1	2.059
	Stage 2	2.102
	Stage 3	2.192
BF4-	Stage 1	0.481
	Stage 2	0.496
	Stage 3	0.649
