# Supplementary material for: Manipulating anion intercalation enables a high-voltage aqueous dual ion battery
Source: Nat Commun. 2021 May 25;12:3106. doi: 10.1038/s41467-021-23369-5 (PMC8149852; doi:10.1038/s41467-021-23369-5)
Supplement: Supplementary file 2 — Description of Additional Supplementary Files [file 41467_2021_23369_MOESM2_ESM.docx]

Description of Additional Supplementary Files

**Supplementary Movie 1**

**Description:** The intercalation process of TFSI^-^ insertion into graphite.
