# Supplementary figures and images for: Manipulating anion intercalation enables a high-voltage aqueous dual ion battery
Source: Nat Commun. 2021 May 25;12:3106. doi: 10.1038/s41467-021-23369-5 (PMC8149852; doi:10.1038/s41467-021-23369-5)

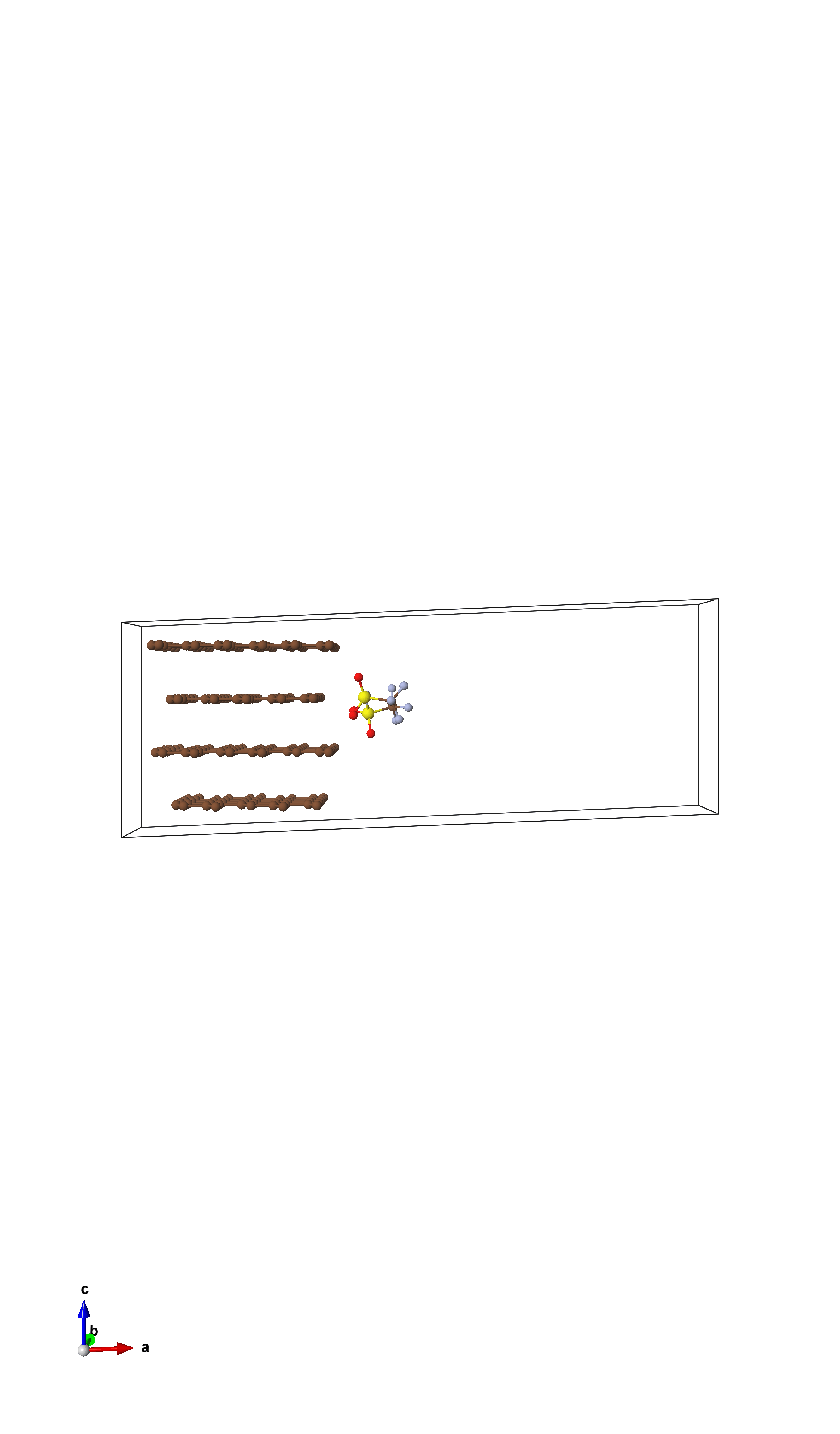

Supplement: Supplementary file 3 — Supplementary Movie 1 [file 41467_2021_23369_MOESM3_ESM.gif]
